# Evolution of the Ferric Reductase Domain (FRD) Superfamily: Modularity, Functional Diversification, and Signature Motifs

**DOI:** 10.1371/journal.pone.0058126

**Published:** 2013-03-07

**Authors:** Xuezhi Zhang, Karl-Heinz Krause, Ioannis Xenarios, Thierry Soldati, Brigitte Boeckmann

**Affiliations:** 1 Department of Biochemistry, Science II, University of Geneva, Geneva, Switzerland; 2 Department of Pathology and Immunology, Central Medical University, University of Geneva, Geneva, Switzerland; 3 SwissProt, Swiss Institute of Bioinformatics, Geneva, Switzerland; 4 Vital-IT, Swiss Institute of Bioinformatics, Lausanne, Switzerland; 5 Center for Integrative Genomics (CIG), Faculty of Biology and Medicine, University of Lausanne, Lausanne, Switzerland; University College Dublin, Ireland

## Abstract

A heme-containing transmembrane ferric reductase domain (FRD) is found in bacterial and eukaryotic protein families, including ferric reductases (FRE), and NADPH oxidases (NOX). The aim of this study was to understand the phylogeny of the FRD superfamily. Bacteria contain FRD proteins consisting only of the ferric reductase domain, such as YedZ and short bFRE proteins. Full length FRE and NOX enzymes are mostly found in eukaryotic cells and all possess a dehydrogenase domain, allowing them to catalyze electron transfer from cytosolic NADPH to extracellular metal ions (FRE) or oxygen (NOX). Metazoa possess YedZ-related STEAP proteins, possibly derived from bacteria through horizontal gene transfer. Phylogenetic analyses suggests that FRE enzymes appeared early in evolution, followed by a transition towards EF-hand containing NOX enzymes (NOX5- and DUOX-like). An ancestral gene of the NOX(1-4) family probably lost the EF-hands and new regulatory mechanisms of increasing complexity evolved in this clade. Two signature motifs were identified: NOX enzymes are distinguished from FRE enzymes through a four amino acid motif spanning from transmembrane domain 3 (TM3) to TM4, and YedZ/STEAP proteins are identified by the replacement of the first canonical heme-spanning histidine by a highly conserved arginine. The FRD superfamily most likely originated in bacteria.

## Introduction

All aerobic living organisms face a dilemma when confronted with the need to assimilate the essential element iron. Indeed, iron is the second most abundant metal on earth, yet the primary form found in the environment is the water insoluble and metabolically inactive ferric ion (Fe^3+^) [1]. The introduction and accumulation of dioxygen, into the ancient oceans and atmosphere, by *Cyanobacteria* completely changed the earth’s initial reductive environment by gradually causing it to become oxidative. As a result, the absorption of bioactive and water soluble ferrous ion (Fe^2+^) became a challenge for all forms of life and left a great impact on evolution [2]. One solution to the dilemma was the emergence of ferric reductases (FRE), which transfer electrons from cytosolic NADPH to extracellular ferric ions to generate the reduced form of ferrous ions, which can then be transported across the plasma membrane by specific iron transporters [3], [4].

Ferric reductases (FRE) and NADPH oxidases (NOX) are homologs [5]. Indeed, three canonical domains are commonly shared by both protein families: a heme-containing 6 transmembrane (6TM) ferric reductase domain and the two C-terminal cytoplasmic FAD-binding and NADPH-binding domains [6]. This common organization most probably reflects the fact that they catalyze similar reactions: Fe^3+^+e^−^ = Fe^2+^ (ferric reductase) and O_2_+ e^−^ = O_2_^−^ (NADPH oxidase).

NOXs transfer electrons to oxygen to produce short-lived superoxide which is the primary reactive oxygen species (ROS), which is then transformed into various other ROS, such as hydrogen peroxide, hypochlorite or ozone [7]. ROS can also be generated as a byproduct in aerobic metabolisms, typically by mitochondria, peroxisomes, chloroplasts, or cytochrome p-450. In contrast, NOXs are devoted to the generation of biologically functional ROS, which play important roles in innate immunity [8], inter/intra-cellular signaling [9], morphogenesis and development [10], [11]. The various physiological and pathophysiological roles of NOX enzymes have been intensively studied and reviewed [7], [12].

Detailed bioinformatics analyses highlighted both the gene phylogeny and the structure of family members [13]–[16]. It was thus shown that ferric reductase domain (FRD) superfamily members exist in a wide variety of organisms, and many species carry multiple gene copies [15]. What is more, structural models have been developed for differing family members and a large number of conserved positions were identified [14]. Various studies inferred the evolutionary relationships of ROS-generating NADPH oxidase families [13]–[15]. Finally, homologs that possess only the conserved ferric reductase domain have been identified in bacteria (YedZ) and eukaryotes (STEAP; Six Transmembrane Epithelial Antigen of Prostate) [16].

In the present study, we characterized the evolutionary history of the FRD superfamily. By adding homologs of species from deep-branching nodes of the species tree, we showed that families and subfamilies emerged earlier than had been previously thought. The gene phylogeny is discussed in the context of structural and functional features. Findings to be highlighted include 1) conserved residues predicted to be crucial for NADPH-oxidases; 2) the probable lateral inheritance of the metazoan STEAP family from bacteria and the synchronous loss of the ancient ferric reductases in this clade; 3) the emergence of the NOX family from EF-hand containing superfamily members; 4) the origin of the FRD superfamily from a bacterial homolog that consists solely of a ferric reductase domain.

## Materials and Methods

### Data Collection

Eukaryotic homologs of the FRD superfamily were obtained from UniProtKB (release 2011_07) [17] [18], by searching – by way of cross-references – for three conserved domains predicted by the Pfam database [19]: the ferric reductase-like transmembrane component (PF01794), the FAD-binding domain (PF08022) and the NADPH-binding domain (PF08030). Further family members were identified in UniProtKB using Blast [20] [21]. The dataset was then complemented with homologs predicted by Ensembl (release 63, 30 June 2011), EnsemblMetazoa (release 10, July 2011), EnsemblPlant (release 10, July 2011), EnsemblFungi (release 10, July 2011), and EnsemblProtist (release 10, July 2011). 47 eukaryotic species were selected as representatives of their corresponding taxonomic groups. Finally, homologs from species of deep-branching nodes of the species tree were added. In order to root the tree, we retrieved homologs from UniProtKB with a conserved ferric reductase domain from complete bacterial proteomes. 29 homologs – including long and short homologs from 16 representative bacteria – were selected for further analysis. The dataset is available in the File S1. For the analysis of the full superfamily phylogeny we retrieved the 2876 predicted ferric reductase domains from Pfam (release 26.0, November 2011).

### Phylogenetic Analysis

Sequences were aligned using MAFFT (version 6) [22] with parameter settings optimized for data with multiple conserved domains and long gaps (E-INS-i) and scoring matrix JTT200 [23]. The gap opening penalty (1.80) and the offset value (0.1) were set above the default. The multiple sequence alignment (MSA) was inspected and edited with JalView (version 2.6.1) [24]. Sequences with long gaps were either replaced by more appropriate isoforms or removed from the alignment. The non-homologous N-terminus of the amino acid sequences was trimmed to the beginning of the conserved ferric reductase domain. From the obtained sequences we constructed a second dataset which included the NOX family members and four members of the DUOX family as an outgroup. Both datasets were realigned as described above. From both datasets we constructed different data models from the conserved regions of the alignments. Following this strategy, we tried to obtain phylogenetic signals at different levels of depth in the gene history. The accuracy of the alignments was evaluated with GUIDANCE (version 1.1) [25] and unreliable columns were removed manually, mostly according to the GUIDANCE score. The best-fit models of evolution were determined with ProtTest (version 3.2) [26] ML phylogenies were calculated for all data models with PhyML 3.0 [27] under the LG amino acid substitution model [28], using eight rate categories which approximated a gamma distribution; the alpha parameter and the proportion of invariable sites were estimated from the dataset. The likelihood was maximized by optimizing the tree topology and branch lengths. The degree of support for internal branches was assessed by the approximate likelihood-ratio test based on the non-parametric Shimodaira-Hasegawa-like procedure (SH-aLRT) as implemented in PhyML.

For the analysis of the superfamily, the sequence redundancy of the predicted ferric reductase domains was reduced to 90% using Jalview. Truncated and dubious sequences were removed and sequences were realigned with MAFFT, using settings optimized for data with one conserved domain and long gaps (L-INS-i) and the scoring matrix JTT200. No other default parameter values were changed. The alignment was edited again with Jalview. This time, the data model was selected in a less stringent way to keep a maximum number of positions for the analysis. Sequence redundancies above 82% were removed from the data model. An ML tree was constructed with PhyML using the same evolutionary model as described above. Phylogenetic trees were inspected and drawn using Archaeopteryx (http://www.phylosoft.org/archaeopteryx).

### Protein Sequence Analysis

All sequences were searched for possible transit peptides (SignalP [29], TargetP [30]), transmembrane domains (TMHMM [31], Phobius [32], MEMSAT [33]), homologous, biased and functional regions (InterPro [34], Pfam [35]), and post-translational modifications (NMT [36]). To avoid over-prediction, we considered positive results only, if 1) a region or site was shown to exist or was analyzed in depth in a previous study for at least one family member; 2) predictors had a high specificity or a region or site is predicted by more than one predictor, or 3) the hit was conserved for the majority of members within a clade.

For the identification of conserved sites within transmembrane domains and conserved adjacent regions, representative homologs of the NOX family (NOX1-4 and co-orthologs from *Fungi*, *Amoebozoa* and *Naegleria gruberi*), NOX-EF group (EF-hand(s) containing members of the NOX group), ppFRE group (protist and plant homologs of the FRE group), and fuFRE (fungal homologs of the FRE group) families, as well as the bacterial short and long forms and the STEAP family were obtained from the UniProt server and previous datasets. Sequences of each family were aligned using MAFFT. The alignments were then inspected manually and sequences that possessed atypical insertions within the transmembrane domains were removed from the MSA. The monophyletic group of YedZ-like proteins was removed from the MSA of bacterial short forms; in the following the group was treated as a group on its own. Well-aligned regions – including the predicted transmembrane domains (TM3–TM5) – were extracted from the alignments. Finally, sequence conservation logos were constructed from the eight MSAs using the WebLogo server (weblogo.threeplusone.com). The alignments used for this purpose included 443 (bacterial short forms), 107 (bacterial long forms), 75 (STEAP), 22 (bacterial short forms similar to bacterial long forms), 123 (fuFRE), 54 (ppFRE), 66 (NOX-EF), and 54 (NOX) sequences, respectively.

The analyses were performed on the Vital-IT High Performance Computing Center (www.vital-it.ch), mafft.cbrc.jp/alignment/server (MAFFT), guidance.tau.ac.il (GUIDANCE), darwin.uvigo.es/software/prottest.html (ProtTest), www.phylogeny.fr [37] (PhyML), www.cbs.dtu.dk/services (SignalP, TargetP, TMHMM), phobius.sbc.su.se (Phobius), pfam.sanger.ac.uk (Pfam), www.ebi.ac.uk/interpro (InterPro), weblogo.berkeley.edu (WebLogo) and local computers.

### Collection of Functional Knowledge

A systematic literature search on experimentally characterized homologs was conducted for NOX homologs and for selected members of the FRE group using PubMed/MEDLINE at www.ncbi.nlm.nih.gov/pubmed and in the reference section of relevant UniProtKB entries.

## Results and Discussion

### Phylogeny of the FRD Superfamily

**Figure 1:**
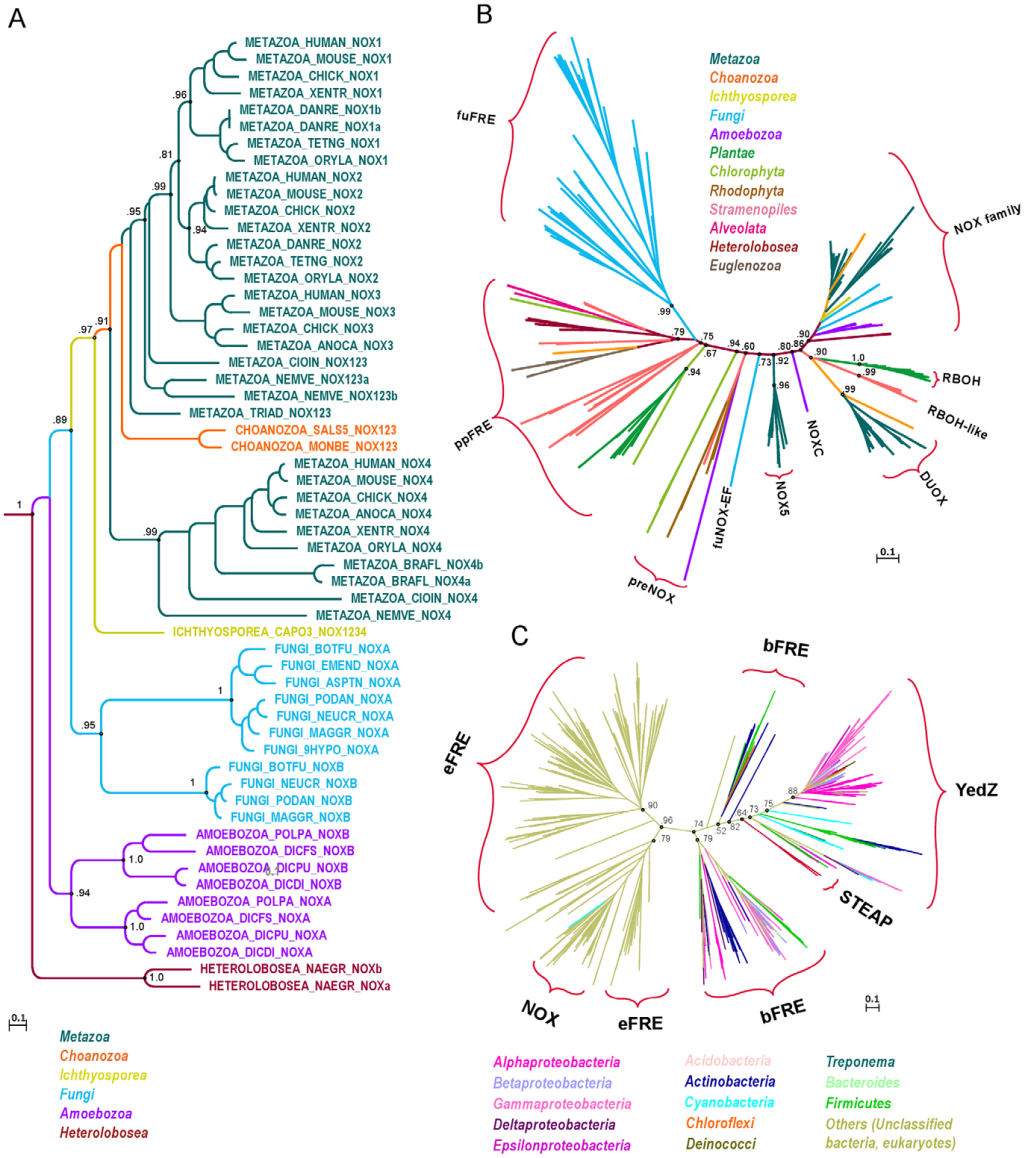
Maximum likelihood phylogeny of the FRD superfamily. A. Phylogram of the NOX family rooted to DUOX genes (outgroup not shown). The tree topology suggests lineage-specific gene duplications in all major taxonomic clades. The NOX1-3 and NOX4 subfamilies possibly diverged before the emergence of metazoans. B. Phylogeny of eukaryotic gene families of the FRE group and the NOX group. According to this model, the DUOX family and NOX family form sister clades, but not the EF-hands containing protein families NOX5 and DUOX. C. Phylogenetic tree of the FRD superfamily. The tree topology proposes that the metazoan STEAP family (red) emerges from the bacterial clade at the base of the YedZ family. The gene AM1_3152 from the cyanobacterium *Acaryochloris marina* (strain MBIC 11017) (UniProtKB: B0CEP3) was probably obtained from an ancestral gene of the eukaryotic NOX5 family. Explanation: The names of gene families and gene groups are indicated with curly brackets. Branch colors correspond to those of the listed taxonomic groups.

**Figure 2:**
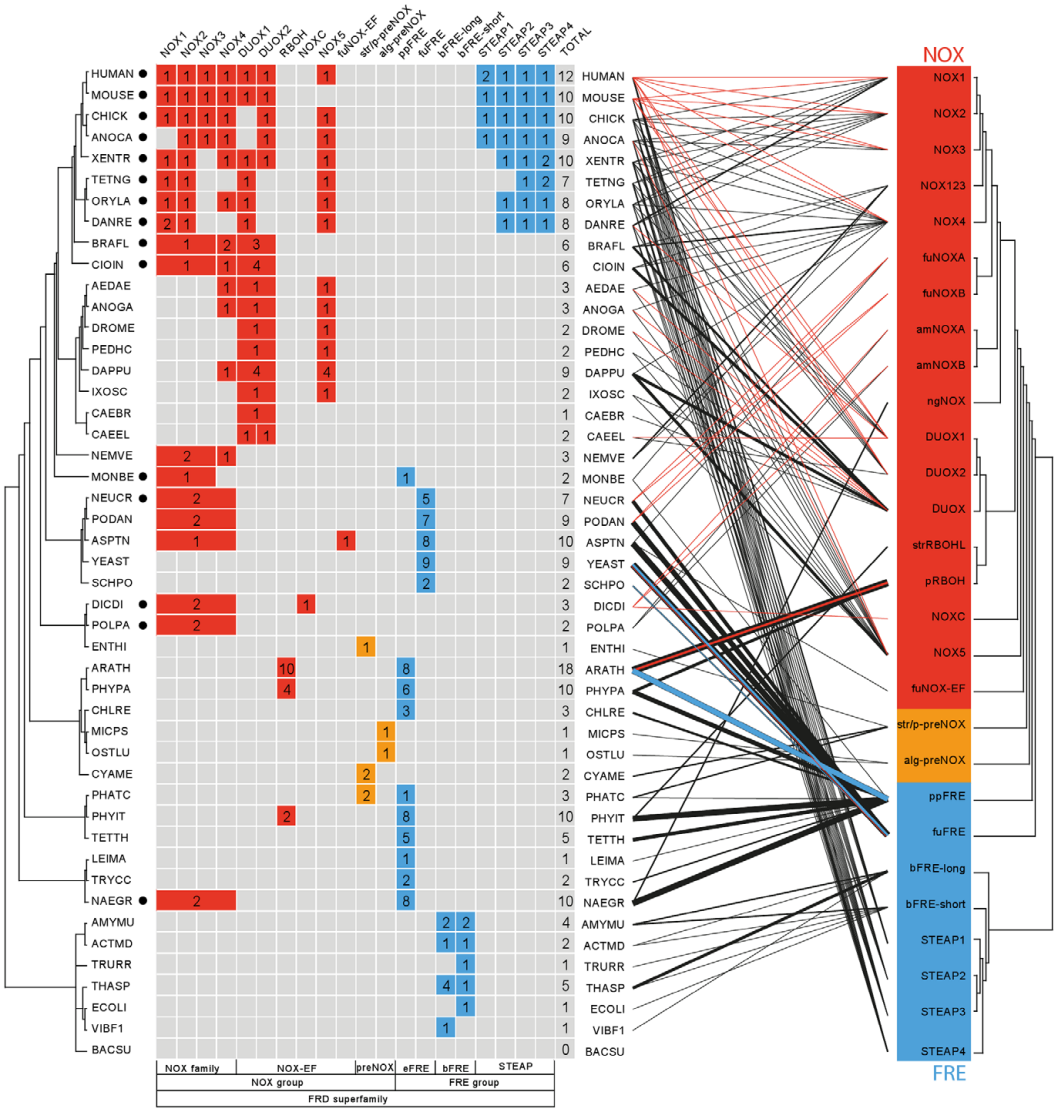
Phyletic profile and molecular function of the FRD superfamily. On the left-hand side, phyletic profile for 47 species: gene copy numbers are plotted in accordance with the species phylogeny (left) and gene families: The number of NOX homologs of a species is given in red cells, FRE homologs in blue cells, and preNOX in orange ones. Some cells are merged according to the family hierarchy. The number of predicted homologs is given in the last column of the phyletic profile. On the right-hand side, gene copies are represented by lines which link the corresponding species and protein families; the thickness of these lines indicates the number of gene copies. Colored lines flag experimentally confirmed gene functions: red = ROS-generating NADPH oxidase activity; blue = metalloreductase activity. Black circles mark species that possess p22phox homologs.

At the highest level of structural organization, members of the FRD superfamily are characterized by a common transmembrane ferric reductase domain (Pfam: PF01794) with structural similarity to cytochrome b of the mitochondrial bc(1) complex. Our analysis initially focused on eukaryotic homologs, whose superfamily members also possess a dehydrogenase module consisting of an FAD-binding domain (Pfam: PF08022) and an NADPH-binding domain (Pfam: PF08030), both oriented towards the cytoplasm. Conserved blocks of these homologous regions account for the data model used in the phylogenetic analyses of the eukaryotic genes; the phylogeny of the FRD superfamily is inferred based on the homologous regions of the ferric reductase domain (Figure 1). The many lineage-specific radiations in the eukaryotic clades throughout evolution are striking (Figure 2, and File S2). Hence, most of the analyzed species possess more than one gene copy of this superfamily. However, organisms do not systematically contain FRD homologs. For 33 out of 263 complete eukaryotic proteomes in UniProtKB (release 2011_12), no homologs with a ferric reductase domain have been predicted until now. These species belong to the taxonomic groups *Alveolata* (18), *Diplomonadida* (3), *Euglenozoa* (2), *Microsporidia* (4), *Parabasalia* (1), *stramenopiles* (1), *Platyhelminthes* (2) and *Arthropoda* (2). For bacteria, FRD homologs are annotated in only 37% of all complete proteomes.

The phylogenetic tree of the FRD superfamily suggests three major protein groups. Clade 1 includes homologs of the ROS-generating protein families (NOX group) as well as homologs of ferric reductases (FRE) predominantly from plants and protists (ppFRE). This group is of special interest in this study, because – based on functionally characterized family members – we presume that the functional shift from a metalloreductase to a ROS-generating protein took place within this branch in an ancestral gene of very early eukaryotes. The phylogeny of the ROS-generating NADPH oxidases (NOX1-5, DUOX, RBOH, preNOX) was analyzed previously [13]–[15]. In agreement with these results are the sub-familial relationships within the NOX family (Figure 1A). The sea anemone *Nematostella vectensis* currently represents the most basal metazoan, and its proteome contains three gene copies, i.e. two ortholog to the NOX1-3 subfamily and one ortholog to the NOX4 subfamily. There is even some evidence that the divergence of these two subfamilies took place prior to the emergence of animal – NOX homologs of the choanoflagellates *Monosiga brevicollis* and *Salpingoeca sp*. (strain ATCC 50818) cluster steadily with the NOX1-3 clade. Furthermore, the NOX family includes the independently duplicated genes from fungi, *Amoebozoa* and *Naegleria gruberi*. The presence of these co-orthologs reveals that a gene of the NOX family was probably already present in very early eukaryotes.

What is more challenging is the interpretation of the evolution of the EF-hand containing protein group (NOX-EF, including NOX5, NOXC, DUOX, and RBOH families). It has been suggested that the EF-hand domain was acquired only once [38]; but, to our knowledge, none of the phylogenetic studies so far have achieved a tree topology that supports a monophyletic origin of NOX-EF prior to the divergence from the NOX family. In our analyses, alternative interfamilial relationships are predicted for the EF-hand containing members when datasets, analysis methods, or analysis parameters are changed (File S3). One explanation for the observed inconsistent topology could be multiple gene duplications that happened close together in time, leaving no or only marginal traces of common evolution in these clades. Because results indicate that the NOX family emerged from the NOX-EF group, we hypothesize that the EF-hand was lost in an ancestral gene of the NOX family.

At the base of clade 1, metalloreductases predominantly from protists and plants (ppFRE) diverge (Figure 1B). Minor clades in between ppFRE and the NOX group are hereafter named preNOX (Figure 1).

Clade 2 includes solely fungal metalloreductases (fuFRE), and the topological placement of this branch outside other eukaryotic metalloreductases is notable (Figure 1B). Our analysis includes genes from five representative fungal proteomes, which possess between two and nine homologs. A more comprehensive analysis of this clade – with homologs from 29 eumycotal proteomes – classified members of the FRE group into 24 families [39].

So far, no metazoan eFRE orthologs have been identified. Indeed, animals possess enzymes with the conserved ferric reductase domain and a ferric reductase activity, but this family (STEAP) is probably xenolog to eFRE (Figures 1C and Figure S4-4 in File S1).

**Figure 3:**
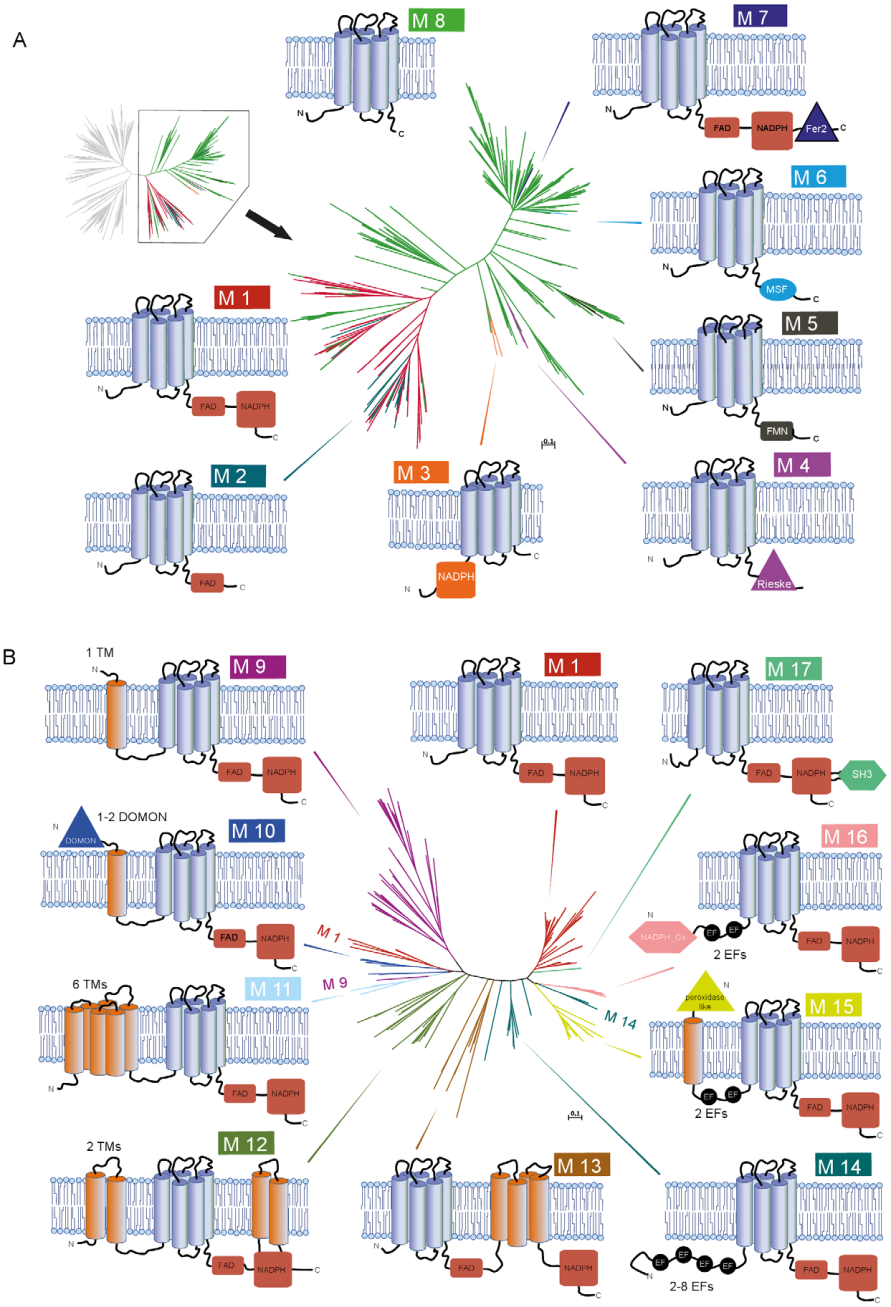
Domain architecture of FRD superfamily members. Models of domain architectures are mapped to the phylogenetic gene trees of bacterial (A) and eukaryotic (B) FRD homologs. Tree branch colors correspond to the color code of the models (see highlight color of model identifiers). The three conserved domains of the ‘eukaryotic structural core’ are colored, and other predicted domains are given in black. Domain forms indicate their function; rounded rectangle = binding of electron donor/hydrogen acceptor: FAD-binding, NADPH-binding (M3), FMN (M4); triangle = electron transfer agent: Ferredoxin/Fer2 (M8), Rieske (M4), DOMON (M10), peroxidase-like domain (M15); circle = regulation of enzyme activity: EF-hands (M14–M16); hexagon = protein-protein interaction: NADPH-oxidase-like domain (M16), SH3 (M17); ellipse = transport of small solutes: MSF (M6).

In order to root the tree, we examined FRD homologs from prokaryotes (clade 3). This clade is composed of bacterial ferric reductases (bFRE) including the bacterial YedZ family and the eukaryotic STEAP family. Bacterial members can be roughly classified into short and long forms; the short forms consist basically of the ferric reductase domain, and the long forms of all three domains conserved in the eukaryotic families (Figure 3). According to Pfam cross-references in UniProtKB (release 2011_12), short forms are about 5.5 times more frequent than the long form. Furthermore, some bacterial proteomes seem to contain only a long form, others only a short form, while some have both forms or none at all (Figure 2). Out of 1471 complete bacterial proteomes provided by UniProtKB, only 543 species possess at least one predicted homolog (Figure S4-1 in File S4). Yet we observed no conspicuous correlation between the bacterial taxonomy and the occurrence of homologs (Figure S4-2 in File S4). During the analysis, we noticed only a few likely cases of HGT from eukaryotes to bacteria. One example is the gene AM1_3152 from the cyanobacterium *Acaryochloris marina (strain MBIC 11017)* (UniProtKB: B0CEP3), which could have been obtained from an ancestral gene of the NOX5 family (Figure 1C). UniProtKB includes four FRE family members from the archaeal domain. A phylogenetic analysis of a prokaryotic dataset reveals that the archaeal genes were probably derived by HGT from bacteria in three independent evolutionary events (Figure S4-3 in File S4).

### Modularity and Functional Diversification

The basic structural component of this superfamily is the transmembrane ferric reductase domain that is present in all the analyzed superfamily members. This domain includes four canonical conserved heme-coordinating histidines in transmembrane domain 3 (TM3) and transmembrane domain 5 (TM5). Their positions in the human NOX2 sequence are 101 and 115 (TM3), and 209 and 222 (TM5). The heme close to the cytoplasm is expected to bind to His-101 and His-209, while the heme close to the cell surface is expected to bind to His-115 and His-222. Most of the bFRD clade homologs consist solely of this domain, but seven distinct modules have been identified, which contribute to the extension of this simple ‘bacterial structural core’ (Figure 3A, model M7). According to Pfam cross-references in UniProtKB, only about 12% of the bacterial homologs have a predicted architecture similar to eukaryotic metal reductases and NADPH oxidases, comprising the transmembrane ferric reductase domain followed by the cytoplasmic FAD-binding and NADPH-binding domains (Figure 3A, model M1). All in all, nine variations of this ‘eukaryotic structural core’ (model M1) were predicted for sequences of our dataset (Figure 3B). Interestingly, additional functional components are generally fused to the N-terminal of the conserved triad. The two C-terminal domains form an elaborated functional unit, in which the NADPH-binding domain provides the enzyme with a readily available electron donor, thereby enhancing its efficiency. Meanwhile the FAD-binding region optimizes the energetic profile of the electron transport chain. This module with redox and electron transfer properties is beneficial to many redox systems, and thus represents an abundant structural compound found in the vicinity of oxidoreductase domains, either as a module in multi-domain enzymes or as a subunit of protein complexes. This structure is often referred to as dehydrogenase domain or dehydrogenase module. While analyzing FRD superfamily members and other unrelated proteins with a dehydrogenase module, transmembrane domain predictors often reported a positive hit in between the two cofactor-binding domains. In all logic, such a protein structure would contradict the module’s biological function. An inspection of known structures (PDB: 3a1f, 1gvh) revealed a hydrophobic region located in the center of the NADPH-binding domain, spanning the C-terminus of a parallel beta-sheet, a short loop opposite the cofactor-binding site and the N-terminal of a proximate alpha-helix. Based on this analysis, both the existence of a transmembrane domain and a hairpin-like structure in between the neighboring domains is therefore unlikely.

In the following sections, the different domain architectures are discussed along with examples of well-studied proteins (Figure 3). An overview of the molecular function of characterized family members is given in Figure 2, and a description of the experimentally confirmed protein functions is available in Table 1.

**Table 1:** Biological functions of eukaryotic FRD superfamily members from published experiments.

Protein, species, UniProtKB identifier	Biological Function	References
**Mammalian NOX1-4**		
NOX1 human: Q9Y5S8, mouse: Q8CIZ9	Signaling (e.g. smooth muscle proliferation, angiogenesis)	[72]–[74]
NOX2 human: P04839, mouse: Q61093	Host defense; signaling to limit inflammation and immune activation	[7] [75]–[78]
NOX3 human: Q9HBY0, mouse: Q672J9	Signaling and/or biosynthesis in the inner ear (otoconia formation)	[79] [80]
NOX4 rat: Q924V1, mouse: Q9JHI8	Signaling (e.g. myofibroblast differentiation, hypoxia response)	[81]–[83]
**Fungal NoxA/B**		
NoxA *Epichloe festucae*: Q2PEP0	Maintain mutualistic status with host plant	[84]
NoxA *Botryotinia fuckeliana*: B0BES1	Maintain pathogenicity; develop penetration structure to infect host plant	[85]
NoxA *Emericella nidulans*: Q8J0N4	Sexual development	[86]
NoxA *Magnaporthe grisea*: A6ZIB7	Maintain pathogenicity; develop penetration structure to infect host plant	[87]
NoxA *Neurospora crassa*: Q7RW00	Female sexual structure formation; asexual development and hyphal growth	[66]
NoxA *Podospora anserine*: B2AA06	Develop penetration structure to infect host plant; degrade host plant cellulose; fruiting body differentiation	[88] [89]
NoxB *B. fuckeliana*: B0BES2	Maintain pathogenicity; colonize in host plant	[85]
NoxB *M. grisea*: A6ZIB8	Maintain pathogenicity; develop penetration structure to infect host plant	[87]
NoxB *N. crassa*: A7UW98	Spores germination	[66]
NoxB *P. anserine*: B2AL10	Ascospore germination; develop penetration structure to infect host plant; degrade host plant cellulose	[88] [89]
**Amoebozoan NOX homologs**		
NoxA *D. discoideum*: Q9XYS3	Development and spore formation	[10]
NoxB *D. discoideum*: Q86GL4	Development and spore formation	[10]
**NOX-EF**		
DUOX1 human: Q9NRD9, mouse: A2AQ92	Thyroid hormone synthesis; mucosal host defense; signaling (e.g. urothelium mechanosensing)	[90]–[96]
DUOX2 human: Q9NRD8, mouse: A2AQ99	Thyroid hormone synthesis; mucosal host defense	[90]–[94] [96] [97]
DUOX *Danio rerio*: F1QVF2	Signaling (chemotaxis, wound healing)	[98]
DUOX *Aedes aegypti*: Q171Q3	Innate immunity; intestinal host defense	[99]
DUOX *Anopheles gambiae*: Q7Q147	Midgut nitration and apoptosis during invasion of *Plasmodium berghei*	[100]
DUOX *D. melanogaster*: Q9VQH2	Innate immunity; signaling (Ca^2+^ channel); protein cross-linking for wing stabilization;epidermal wound healing	[11] [101]–[103]
DUOX1 *Caenorhabditis elegans*: O61213	Innate immunity; host defense; protein cross-linking in cuticular extracellular matrix	[104]–[106]
DUOX *Lytechinus variegatus*: Q5XMJ0	Protein cross-linking in fertilization envelope	[107]
RBOHC *Arabidopsis thaliana*: O81210	Signaling (Ca^2+^ channel); root cell elongation	[108]
RBOHD *A. thaliana*: Q9FIJ0, RBOHF:*A. thaliana*: O48538	Host-pathogen interaction; signaling (e.g. ROS as second messengers in abscisic acidsignaling in guard cells)	[109]–[112]
NOX5 human: Q96PH1	Signaling (e.g. prostate cancer cells, spermatocytes, marginal B lymphocytes)	[113]–[116]
NOX5 *A. gambiae*: Q7PNG0	Midgut epithelial nitration and innate immunity	[117]
NOXC *D. discoideum*: Q54F44	Development and spore formation	[10]
**Plant eFRE homologs**		
FRO2 *A. thaliana:* P92949	Fe^3+^ reduction/acquisition in root surface; iron and copper homeostasis; chilling stresstolerance (block ROS signaling during chilling)	[3] [118]–[120]
FRO3 *A. thaliana:* F4I4K7	Fe^3+^ reduction/acquisition in root vascular cylinder and shoots (mitochondria); iron and copper homeostasis	[120] [121]
FRO4 *A. thaliana:* Q8W110	Iron and copper homeostasis	[122]
FRO5 *A. thaliana:* Q9FLW2	Iron and copper homeostasis in root	[120] [122]
FRO6 *A. thaliana:* Q8RWS6	Fe^3+^ reduction in shoots (chloroplast); iron and copper homeostasis	[120] [121] [123]
FRO7 *A. thaliana:* Q3KTM0	Fe^3+^ reduction/acquisition in chloroplast for photosynthesis; iron and copper homeostasis	[48] [120]
FRO8 *A. thaliana:* Q8VY13	Iron and copper homeostasis in leaves	[120] [121]
**Fungal eFRE homologs**		
FRE1 *S. cerevisiae:* P32791	Fe^3+^ and Cu^2+^ reduction/acquisition; iron and copper homeostasis	[44] [124] [125]
FRE2 *S. cerevisiae:* P36033	Fe^3+^ and Cu^2+^ reduction/acquisition; iron and copper homeostasis	[124] [126]
FRE3 *S. cerevisiae:* Q08905	Fe^3+^ reduction; iron homeostasis	[46] [124]
FRE4 *S. cerevisiae:* P53746	Fe^3+^ reduction; iron homeostasis	[46] [124]
FRE5 *S. cerevisiae:* Q08908	Iron homeostasis	[46]
FRE6 *S. cerevisiae:* Q12473	Export iron and copper from vacuole; iron and copper homeostasis	[46] [127] [128]
FRE7 *S. cerevisiae:* Q12333	Copper homeostasis	[46]
AIM14 *S. cerevisiae:* P53109	ROS generation; apoptosis; actin cable formation	[47]
FRP1 *S. pombe*: Q04800	Fe^3+^ reduction/acquisition; iron homeostasis	[129]

### bFRD: Extensions to a Simple Module

**Figure 4:**
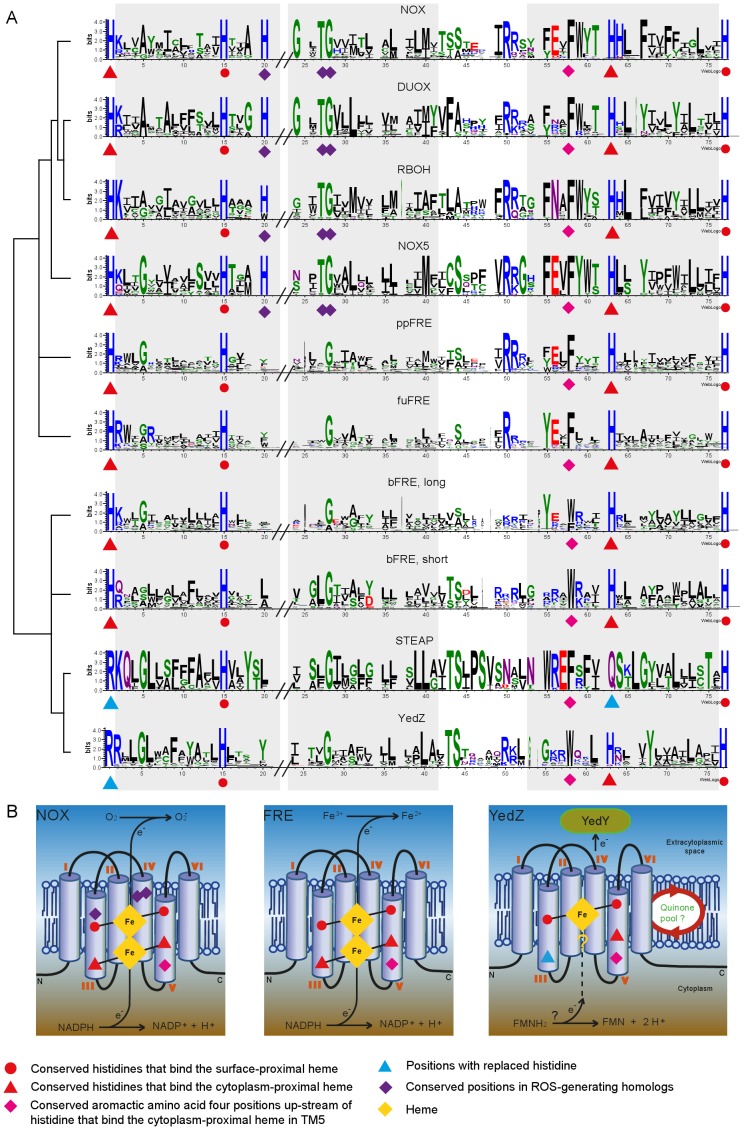
Sequence conservation logos and the proposed structure of the ferric reductase domain of protein groups from the FRD superfamily. A. Transmembrane domains TM3 to TM5 - as predicted for human cytochrome b-245 heavy chain (NOX2) - are indicated by gray rectangles. The cladogram indicates the phylogenetic relationship of the analyzed homologous groups. In the conservation logos, the height of the stacks indicates sequence conservation; the width of the stacks is proportional to the fraction of amino acids, thus narrowed within gapped regions. B. Proposed structure of ferric reductase domain with conserved amino acid residues corresponding to the annotation in figure 4A.

Typical representatives of the ‘one-domain’ homologs from bacteria are members of the YedZ family found in *Proteobacteria,* which are involved in redox regulation and transmembrane electron transfer [40]. The *E.coli* homolog of this family has been shown to form an oxidoreductase complex with the soluble catalytic molybdoenzyme YedY [41]. Out of 1,755 currently available bacterial homologs in UniProtKB, YedZ is – to our knowledge – the only characterized family member. bFRD homologs are probably involved in many different biological processes, as can be inferred from the diverse fusion proteins. Six modular extensions to the bacterial structural core have been predicted for members of the bacterial clade and, as a matter of fact, all these domains are related to functions of electron transport and/or exchange. Structural modifications are also observed in homologs of the eukaryotic STEAP family that emerged from the bacterial FRE branch. Like its bacterial homologs, STEAP1 proteins consist only of a ferric reductase domain but its paralogs, STEAP2-4, possess additionally an NADPH-binding domain (‘NADP oxidoreductase coenzyme F420-dependent’ domain; Pfam: PF03807) in their N-terminal region. Such a domain architecture has not been predicted for bacterial metal reductases. STEAP family members have been shown to possess both ferric reductase and cupric reductase activities [42] [43]. Thus even members of the FRD superfamily, which do not have the three canonical domains, appear to be involved in the same molecular function, but probably using different reaction mechanisms. We compared the conserved amino acids found in the transmembrane domains of both the short and the long forms of bFRD. This revealed that most short forms possess only two (STEAP2-4: His-115, His-222) or three (YedZ, STEAP1: His-115, His-209, His-222) of the conserved histidines and thus probably bind only the surface-proximal heme (Figure 4). Experimental evidence for functional electron transport through the membrane via a single heme is scarce but some evidence has been reported for YedZ [41]. Instead of the cytoplasm-proximal heme-binding histidines we found a well-conserved arginine (TM3) and glutamine (TM5). In contrast, the long forms possess all four conserved histidines. The few short forms that contain all four conserved histidines are found in the direct vicinity of the long forms in our phylogenetic tree. This finding supports the tree topology in that STEAP is more close related to the bacterial short form, and bacterial long forms are more closely related to eukaryotic homologs.

Most structural modifications are found in orthologs of closely related species, thus indicating that variations of the basic core are generally not persistent in bacteria, a fact which is typical for the evolution of prokaryotic proteins. Hence, the enduring bacterial ‘long form’ might be more efficient than functional equivalents or could have a unique function.

### eFRE: Variations in ‘Transmembrane’

Most, if not all, fungal metalloreductases (fuFRE) contain an additional predicted transmembrane domain that precedes the three conserved domains of the eukaryotic structural core. The nine *Saccharomyces cerevisiae* homologs are among the best-studied examples of this group. As they are important components of the high affinity uptake system for iron and copper ions, FRE1 and FRE2 are involved in both ferric and cupric ion reduction. There is evidence that FRE3-6 homologs are specific iron reductases, whereas the FRE7 homolog is a specific copper reductase [44]–[46]. A recent study shows that yeast YNO1 (AIM14) - one of the 9 yeast FREs - generates ROS and affects both apoptosis and actin cable formation [47]. The function of the N-terminally fused transmembrane region is unknown.

In some ways similar to the fungal metalloreductases, the majority of protist and plant homologs (ppFRE) in this group bear predicted transmembrane regions in addition to those of the ferric reductase domain. Two or six transmembrane domains are found in the N-terminal of these homologs, but additional transmembrane helices are also predicted close to the C-terminus, within the NADPH-binding domain in clade members from *Viridiplantae* and *stramenopiles*. A well-studied example is the tissue-specific expression of the eight paralogs of the ferric chelate reductase (FRO) family from *Arabidopsis thaliana* [3]. Three of these homologs are located in the membrane of organelles: FRO7 in chloroplasts, FRO3 and FRO8 in mitochondria [48] [49]. Nevertheless, all these gene copies emerge from the eukaryotic clade and it is therefore unlikely that they originate from an organelle genome.

Besides additional transmembrane domains, a single clade of the eFRE group has acquired a new structural module. Members of a subfamily – including seven paralogs from the *Heterolobosea N. gruberi* and homologs from the stramenopiles *Albugo laibachii* (UniProtKB: F0X089) and *Phytophthora infestans* (UniProtKB: D0MUZ3) – contain one or two extracellular DOMON domains (Pfam: PF03351). The DOMON domain was predicted to possess a hydrophobic pocket that binds heme and sugar [50] [51]. It has also been suggested that it participates in an electron-transfer system [50]. A signal peptide was predicted only for a homolog presenting two DOMON domains.

Note that no FRE-EF forms were observed in bacteria and eukaryotes, suggesting that the regulatory EF hand domains were acquired by superoxide-producing NOX enzymes.

### preNOX and NOX-EF: Emergence of ROS Generation and Activity Regulation

At the base of the NOX group, homologs from *Rhodophyta*, *stramenopiles* and *Viridiplantae* contain four predicted additional transmembrane helices between the FAD-binding and NADPH-binding domains. One study revealed a potential ROS-generating NADPH oxidase activity for the homolog of the red algae *Chondrus crispus*, [52]. This experimental evidence is interesting, because with the occurrence of EF-hands at the N-terminus, such homologs are known or expected to produce ROS for specific biological functions. The algae, however, diverged before this domain was acquired.

The functional shift from metalloreductases to ROS-generating NADPH oxidases probably occurred early on in the NOX group and, to some extent, seems to be linked to the occurrence of EF-hands in the N-terminus. The binding of Ca^2+^ to EF-hands can cause conformational changes linked to regulatory functions, as for instance in calmodulin [53]. A need for regulated ROS production is plausible because of the toxicity of the products, as illustrated by their function in oxidative innate immune defense. At least one EF-hand was predicted for all members of the NOX-EF group. As the biologically active structure is generally a pair of EF-hands, and not all predicted EF-hands have the capacity to bind Ca^2+^, representative family members were inspected manually (Cox JA, personal communication). All the studied homologs possessed at least two EF-hands, of which at least one was canonical and hence assumed to be Ca^2+^-binding. Fungal members of the paraphyletic group of NOX-EF have a single Ca^2+^-binding EF-hand motif. They seem to exhibit ROS-generating NADPH oxidase activity and have been suggested to be regulated by Ca^2+^ [15] [54] [55]. The metazoan NOX5 family members contain four EF-hands, and studies in human and mouse confirmed Ca^2+^-regulated ROS-generation [56] [57].

A comparison of the eFRE metal reductases with expected NADPH oxidases indicated two positions that are conserved in members of the latter group: a histidine in addition to the two heme-binding histidines in transmembrane domain 3 (TM3), and a conserved threonine in transmembrane domain 4 (TM4) (Figure 4). In accordance with their sequence position in human NOX2, we refer to these sites as His-119 and Thr-178. His-119 is located within the alpha-helix close to the cell surface. Well-studied heme-binding proteins involved in oxygen-transport are hemoglobin and myoglobin. In both molecules, dioxygen-binding is stabilized by a hydrogen bond to a conserved histidine residue distal to the heme-binding sites [58]. We propose that a similar stabilizing mechanism involving His-119 could apply to the ROS-generating homologs of the superfamily, as this position is conserved in the vast majority of the analyzed homologs of the NOX group. Exceptions were found in species with multiple NOX inparalogs, and each of these species’ proteomes presented at least one gene copy that possessed the conserved histidine (His-119). One of these exceptions is NOXC of *D. discoideum*. Preliminary studies using a NOXC knockout strain showed reduced levels of stimulated ROS generation in vegetative and starved cells (Zhang X, Soldati T, unpublished data). We therefore assume, that the second conserved residue, Thr-178, is equally important for NADPH oxidase activity, but a gating function seems more likely than oxygen-binding. According to an analysis of the functional roles of catalytic residues [59], threonine has been found to predominantly perform stabilizing functions. Of note is a conserved glycine residue (Gly-179) adjacent to the conserved threonine, which is actually not specific to NOX homologs, but which forms a conserved dipeptide in the probable ROS-generating proteins. Natural variants in human NOX2 have been identified in patients with inheritable disorders related to innate immunity: mutations in His-119 as well as in Gly-179 lead to a chronic granulomatous disease (CGD) phenotype [60] [61], and mutations in Thr-178 to Mendelian susceptibility to mycobacterial disease (MSMD) syndrome phenotype [62]. In summary, four conserved positions are probably indicative in distinguishing ROS-generating homologs of the NOX group from presumptive metalloreductases: His-101, His-119, Thr-178 and Gly-179.

These findings are key in identifying the point of functional divergence in evolution. The preNOX group diverges at the very base of the NOX group, and includes genes from stramenopiles, red algae as well as green algae and fungi. Except for the fungal genes, all these members lack His-119; green algae even lack the second heme-binding histidine in TM3, but all homologs possess the conserved dipeptide. Hence, the homologs from *stramenopiles* and rhodophytes – but not the genes of the chlorophytes analyzed here – are likely to possess a ROS-generating activity that is not regulated by Ca^2+^. All other homologs of the NOX-EF group possess two or more EF-hands. In addition, two other structural components are found in members of this group: a domain of unknown function that encompasses the N-terminus of the NADPH oxidase (NADPH-oxidase Ox; Pfam: PF08414) and the heme-containing peroxidase-like domain (Pfam: PF03098). These two additional domains are the hallmark of two separate NOX families. The cytoplasmic NADPH-oxidase domain is attached to the N-terminus of the EF-hands in the RBOH family of land plants. Interestingly, closely related homologs from stramenopiles (RBOH-like) seem to possess EF-hands, but show no predicted domains at the N-terminus. Members of the DUOX family comprise up to three predicted EF-hand motifs, and a further transmembrane domain links to an extracellular peroxidase-like domain. The predicted signal peptide might be required to translocate the peroxidase-like domain to the extracellular space. Members from both families have been biochemically characterized as ROS-generating and Ca^2+^-regulated enzymes, strongly suggesting a conserved regulation pattern within the NOX-EF group (see Table 1).

### The NOX Family: ROS Generation and Increasing Complexity of Regulation

Analyses of the extremely divergent domain architectures suggest that two distinct series of events appear to have led to an increased complexity of regulation of ferric reductase domain enzymes. First, as detailed in previous sections, during the early evolution of the superfamily, NOX enzymes acquired longer N-termini with various regulatory and functional domains. Second, the N-terminus was lost subsequent to the gene duplication event that gave rise to the emergence of the NOX1-4 family, which includes the meatozoan NOX1-4 homologs, and the independently duplicated co-orthologs of fungi (NOXA, NOXB), *Amoebozoa* (NOXA, NOXB) and *Naegleria*. The loss of the EF hand-containing N-termini occurred concomitantly with the emergence of separate cytosolic and membrane bound regulatory subunits. An advantage of multi-domain proteins is the colinear expression of functional units that are essential for a specific cellular task, bypassing the need for coordinated expression of separate gene products. However, a more fine-grained regulation is possible when multiple differentially expressed and regulated subunits assemble into a functional complex.

Developmental steps to a higher regulation complexity from NOX4, to NOX3, NOX1 and NOX2 can be traced in the NOX family. Of all these, the best studied NOX ortholog is NOX2 and its regulatory network has shown to be particularly complex. Indeed, its fine regulation requires an interaction both with the membrane component p22phox and the cytosolic components p47phox, p67phox, and p40phox. Finally, it also associates with a Rac GTPase that is responsible for the activation of the protein complex [15]. As a paradigm for the regulation of this class of enzymes, in the resting state of human NOX2, the proline rich region (PRR) at the N-terminus of p47phox interacts with the N-terminal SH3 domain of p67phox, while the PB1 domains of p67phox and p40phox interact with each other. Upon stimulation, GTP-bound Rac interacts with the N-terminal region of p67phox. Due to conformational changes in these cytosolic components, the SH3 domain of p47phox is able to interact with the N-terminal PRR of p22phox, which will further interact with NOX2 and activate its enzymatic function [15]. Only four regulators are needed for the function of NOX1: namely p22phox, the p47phox homolog NOXO1 (NOX1 organizer), the p67phox homolog NOXA1 (NOX1 activator), and Rac [15]. It is remarkable that most of these subunits are paralogs of the NOX2 regulators, stressing the importance of differential regulation. In contrast to this, the organization around NOX3 and NOX4 seems to be less tight. Indeed, NOX3 activity is up-regulated either by NOXO1 or by NOXA1, but only p22phox is required for ROS production [63]. NOX4, the most distant paralog to NOX2, requires only p22phox for ROS generation and seems to function independently of further regulators [15] [64].

Precursors of the domains that interact with these essential regulatory subunits can already be found in the NOX homologs of unicellular organisms. The two orthologs of *Heterolobosea N. gruberi*, for instance, contain an SH3 domain (Pfam: PF00018) within their C-terminal NADPH binding domain. Usually, the SH3 domain mediates protein complex assembly via binding to proline rich regions (PRR) of binding partners [65]. Thus, the SH3 domain found within the NADPH-binding domain of *N. gruberi* NOX homologs suggests that some unknown PRR-containing protein partner is involved in their regulation [15]. Interestingly, an SH3 domain has been identified in the human p67phox, but, alternatively, the homolog of the social amoeba *Dictyostelium discoideum* carries a functionally analogous WW domain for protein-protein interactions (Pfam: PF00397) [10]. Furthermore, the N-terminus of both homologs consist of four TPR repeats, (Pfam: PF00515), a feature also conserved in fungal NOX regulators (NOXR) found in *Pezizomycotina*, including *Aspergillus nidulans, Neurospora crassa* and *Epichloe festucae* [66] [67]. This conserved region mediates interaction with small Rac GTPases, thereby linking these NOX enzymes directly to the regulation by Rho family members [68]. Although it is still not clear which of the many Rac homologs might be involved in the regulation of *D. discoideum* NOXA and NOXB, Rac1A, -B and -C show the greatest homology with Rac2 in human neutrophils. In addition, all of the Rac2 residues at the interface with p67phox are conserved, suggesting that a similar activation process might also have occurred in *D. discoideum* [10].

### Functional Congruence in Domain Composition, Sequence Divergence at Functional Sites

Domain architectures evolve at different modes in prokaryotes and eukaryotes and our results are characteristic for the two superkingdoms. Prokaryotic proteins generally possess a less complex domain composition than eukaryotes, but undergo fusion and fission events more frequently. Hence, proteins with a more complex domain organization are found in small clades (Figure 3). In contrast, eukaryotic proteins tend to be composed of more domains, but structural alterations can occur stepwise and at a low rate. Thus, to some extent it is possible to trace back evolution based on the continuous increase of structural complexity in proteins from related species. In the case of the FRD superfamily, this leads to a higher structural diversity in eukaryotic homologs than prokaryotic ones. Despite the differing mode of evolution regarding the domain architecture, congruent evolution can be observed with respect to the function of domains that enlarge the bacterial and eukaryotic structural core. First of all, it is likely that the bacterial long forms and the equivalent eukaryotic structural core result from independent fusion events. Furthermore, four distinct domains serve as electron transfer agents (Ferredoxin (Fer2), Rieske, DOMON, peroxidase-like domain) and two distinct domains assist protein-protein interactions (NADPH-oxidase-like domain, SH3). Thus, the reductive function of the ferric reductase domain was most probably conserved over more than two billion years. This assumption is supported by the conservation of the surface-proximal heme-binding histidines in TM3 and TM5. Finally it is notable that domains with equivalent functions can occur on either side of the membrane.

At the sequence level, divergent evolution can be observed at functional sites, which are assumed to reflect changes in substrate selectivity and possibly the reaction mechanism. The functional shift from metal reductase to NADPH oxidase activity probably occurred early on in evolution at the base of the NOX clade. Four amino acids have been identified to distinguish metalloreductases from ROS-generating NADPH oxidases. The replacement of the first canonical heme-spanning histidine by a highly conserved arginine in members of the prokaryotic YedZ family and the eukaryotic STEAP family may indicate a change in the reaction mechanism.

### Origin of the FRD Superfamily

**Figure 5:**
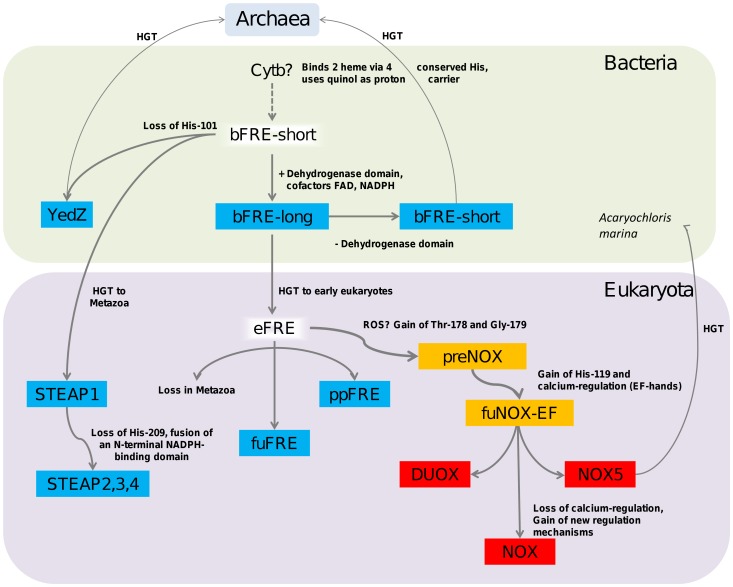
A model of the evolutionary history of the FRD superfamily. The ancestral system may have used reduced quinol to produce soluble ferrous ions and progressed into a highly regulated system that generates immunologically potent ROS by using NADPH as electron source.

The ferric reductase domain may well have emerged from cytochrome b. The phylogeny of the FAD-binding domain suggests the fusion of a ferric reductase domain to a dehydrogenase module (File S5). Based on the likely absence of ancient ferric reductase domain gene copies in archaeal genomes, it seems reasonable to expect a bacterial origin of the ferric reductase domain. However, as shown above, only about 37% of all bacterial genomes possess a homolog of the FRD superfamily. Supporting arguments for a bacterial origin of this superfamily are – in particular – the presence of homologs in a broad variety of taxonomic groups compounded by the fact that the ferric reductase activity is only essential for obligate aerobic species that have no access to ferrous ion resources and possess no non-reductive iron uptake system. Furthermore, as a typical operational gene, ferric reductase is not only transferred more easily between bacteria [69], but is also likely to descend from bacteria early on in eukaryotic evolution [69]–[71]. Our phylogenetic superfamily tree shows a bacterial and a eukaryotic clade, suggesting a unique event of ancient gene transfer between the superkingdoms. The model is summarized in Figure 5.

### Conclusions

This study reveals that the ferric reductase domain superfamily probably originated from a short bacterial homolog that consisted solely of a ferric reductase domain. During evolution these ‘one-domain’ homologs were extended by different modules that either participate in redox systems or are regulatory components. The C-terminal fusion of an FAD- and NADPH-binding domain probably resulted in the core of the long homologs, which are mostly found in eukaryotes. Phylogenetic analysis reveals that the highly diverse number of gene copies per species is derived by extensive lineage-specific gene gain and gene loss throughout evolution. The functional shift from metal reductase to NADPH oxidase activity probably occurred early in evolution at the base of the NOX clade. The generation of potentially toxic ROS is accompanied by increased complexity of regulatory systems. One such regulatory component is p22phox, which appears with the emergence of the NOX family; its origin is still unknown.

Four amino acids have been identified to distinguish metalloreductases from ROS-generating NADPH oxidases. We believe that it might be possible to detect further sites for the construction of even more specific function predictors based on the comparison of clade-specific conservation signatures. Finally, we plan to use conservation signatures for the revision of unresolved nodes. Preliminary results suggest taking the option of classifying the superfamily into three main groups, namely NOX, FRE and YedZ/STEAP. These and other related issues will be the subject of future interesting studies.

## Supporting Information

File S1 **Sequence data and domain predictions.**
(XLSX)

File S2 **Maximum likelihood phylogeny of eukaryotic gene families of the FRE group and the NOX group.** The phylogenetic tree is a detailed presentation of Figure 1B. Tree branches are colored according to the taxonomic classification of the species.
(TIF)

File S3 **Exploration of the tree space.** Phylogenies of the FRD superfamily from multiple analyses. Gene families are colored in the phylogenetic tree and family names are given. Branch support values of major internal nodes are indicated.
(PDF)

File S4 **Analysis of bacterial FRD superfamily members.**
(PDF)

File S5 **Evolution of the FRD superfamily from the view of its core domains.**
(PDF)
