# Supplementary figures and images for: Evolution of the Ferric Reductase Domain (FRD) Superfamily: Modularity, Functional Diversification, and Signature Motifs
Source: PLoS One. 2013 Mar 7;8(3):e58126. doi: 10.1371/journal.pone.0058126 (PMC3591440; doi:10.1371/journal.pone.0058126)

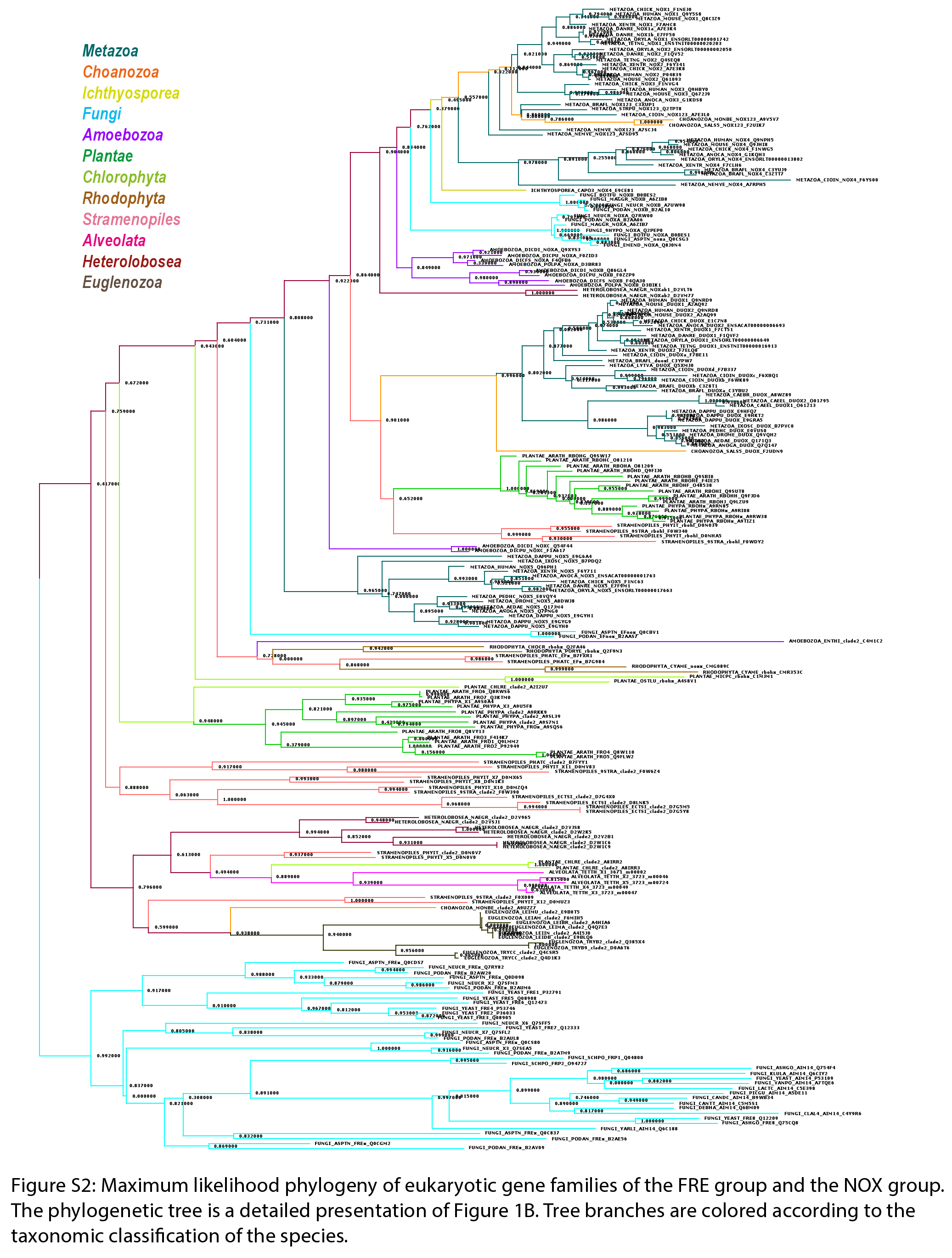

Supplement: File S2 — Maximum likelihood phylogeny of eukaryotic gene families of the FRE group and the NOX group. The phylogenetic tree is a detailed presentation of Figure 1B. Tree branches are colored according to the taxonomic classification of the species. (TIF) [file pone.0058126.s002.tif]
