# Supplementary material for: Evolution of the Ferric Reductase Domain (FRD) Superfamily: Modularity, Functional Diversification, and Signature Motifs
Source: PLoS One. 2013 Mar 7;8(3):e58126. doi: 10.1371/journal.pone.0058126 (PMC3591440; doi:10.1371/journal.pone.0058126)
Supplement: File S3 — Exploration of the tree space. Phylogenies of the FRD superfamily from multiple analyses. Gene families are colored in the phylogenetic tree and family names are given. Branch support values of major internal nodes are indicated. (PDF) [file pone.0058126.s003.pdf]

File S3: Exploration of the tree space. Phylogenies of the FRD superfamily from multiple analyses. Gene families are colored in the phylogenetic tree and family names are given. Branch support values of major internal nodes are indicated.

Figure S3-1. ML phylogeny of the FRD superfamily: 1286 genes; PhyML, WAG+F+gamma(8)+I, SH aLRT branch support.

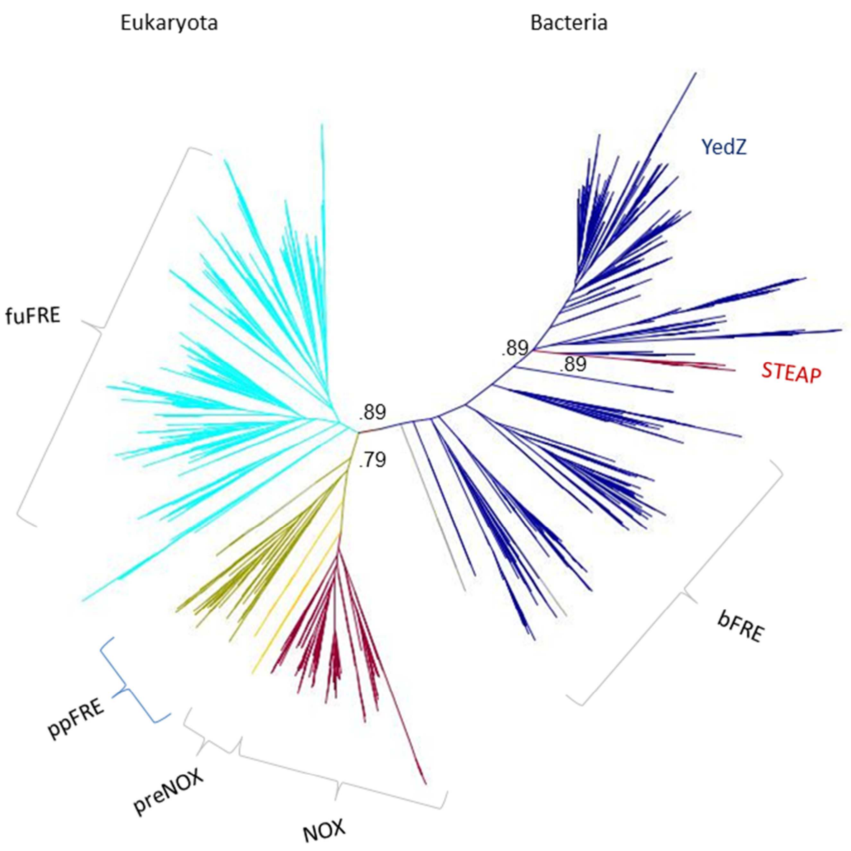

Figure S3-2. BI phylogeny of the FRD superfamily: 198 genes, data model: 171 amino acids; MrBayes, WAG+F+gamma(8)+I, 5m generations, branch support: posterior probabilities.

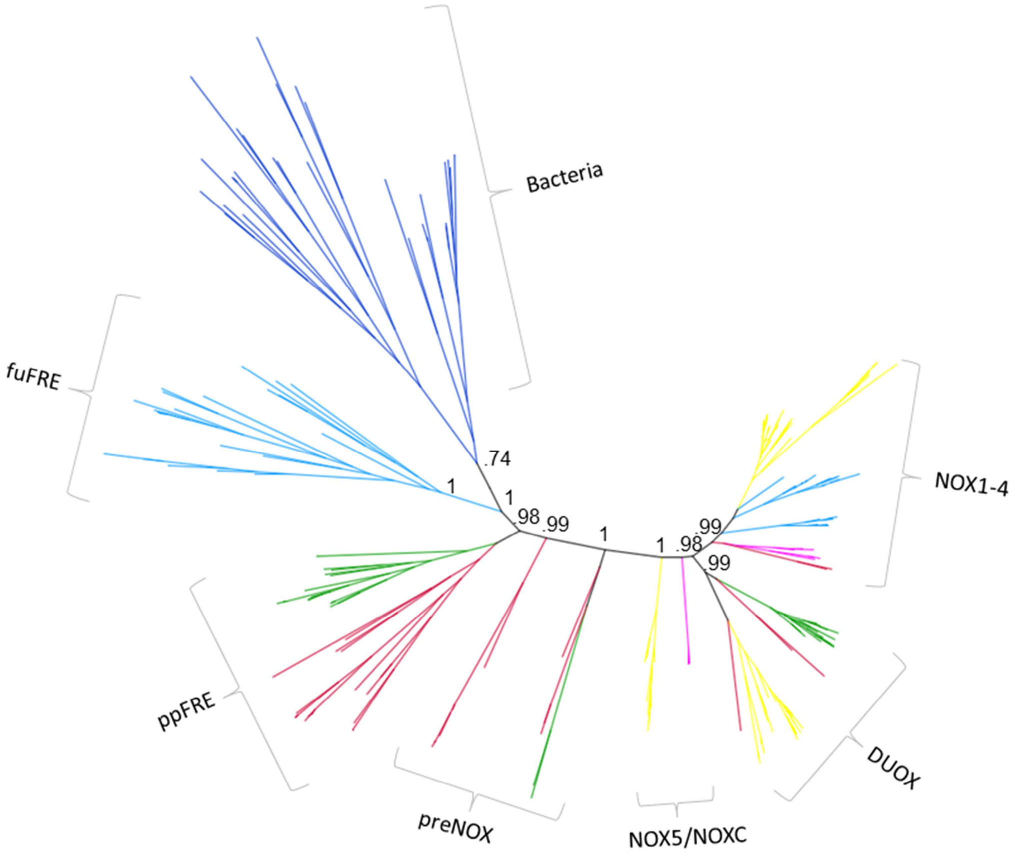

Figure S3-3 A-E. NOX-EF: EF-hand-containing families of the NOX group; branch support values for the clades of DUOX/RBOH and NOX5/NOXC are indicated. The topology shown in B, C and D is the most representative.

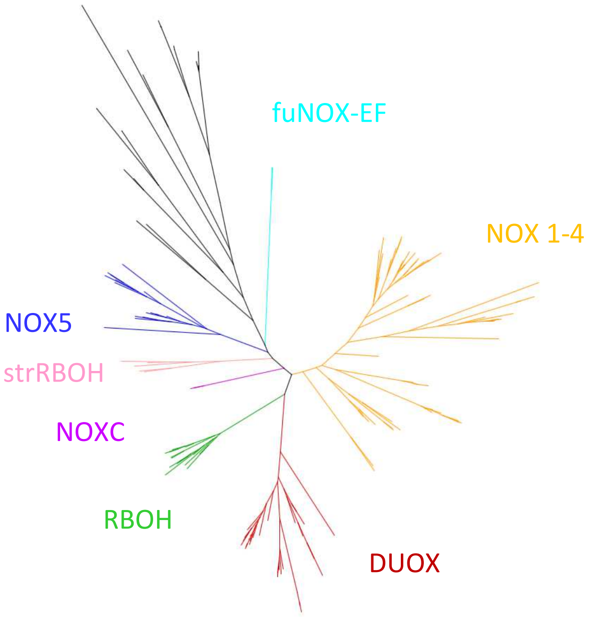

A

ML phylogeny of the NOX group: 148 genes, data model: 264 amino acids, PhyML, WAG+F+gamma(8)+I.

Branch support (aLRT-SH): DUOX/RBOH: 0.87, NOX5: 0.71, fuNOX-ef: 0.93.

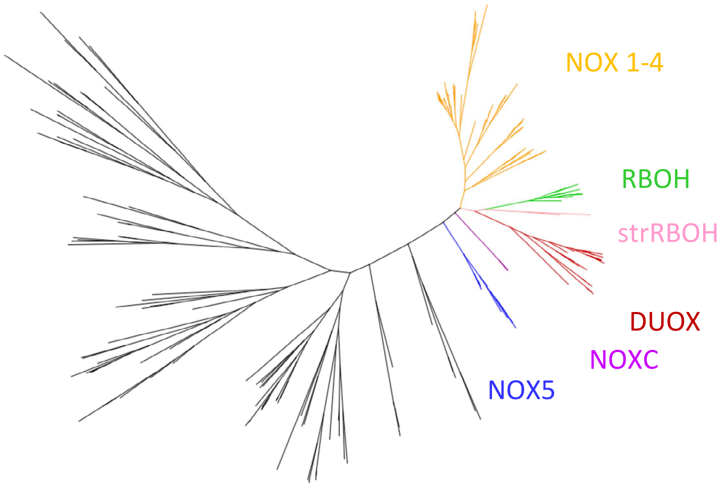

B

BI phylogeny of the FRD superfamily: 198 genes, data model: 171 amino acids,MrBayes, WAG+F+gamma(8)+I, 5m generations.

Branch support, posterior probabilities: DUOX/RBOH: 0.83, NOX5: 1, fuNOX-ef: NA.

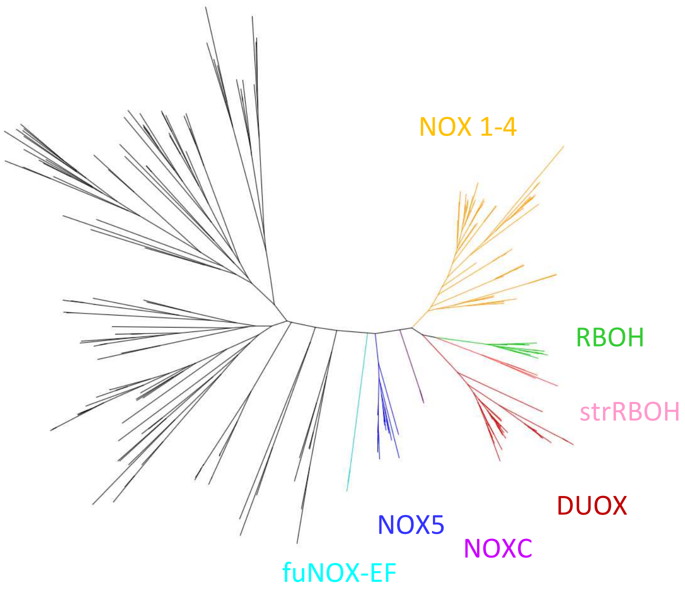

C

ML phylogeny of the FRD superfamily: 240 genes, data model: 156 amino acids, PhyML, WAG+F+gamma(8)+I.

Branch support, aLRT-SH: DUOX/RBOH: 0.90, NOX5: 0.48, fuNOX-ef: 0.91.

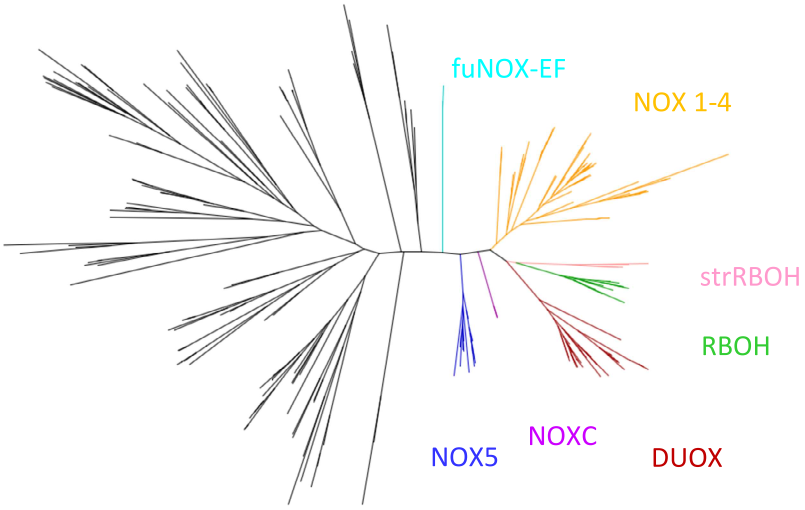

D

ML phylogeny of the FRD superfamily: 240 genes, data model: 156 amino acids, RAxML, WAG+F+gamma(8)+I.

Branch support, bootstrap: DUOX/RBOH: 44, NOX5: 45, fuNOX-ef: 51.

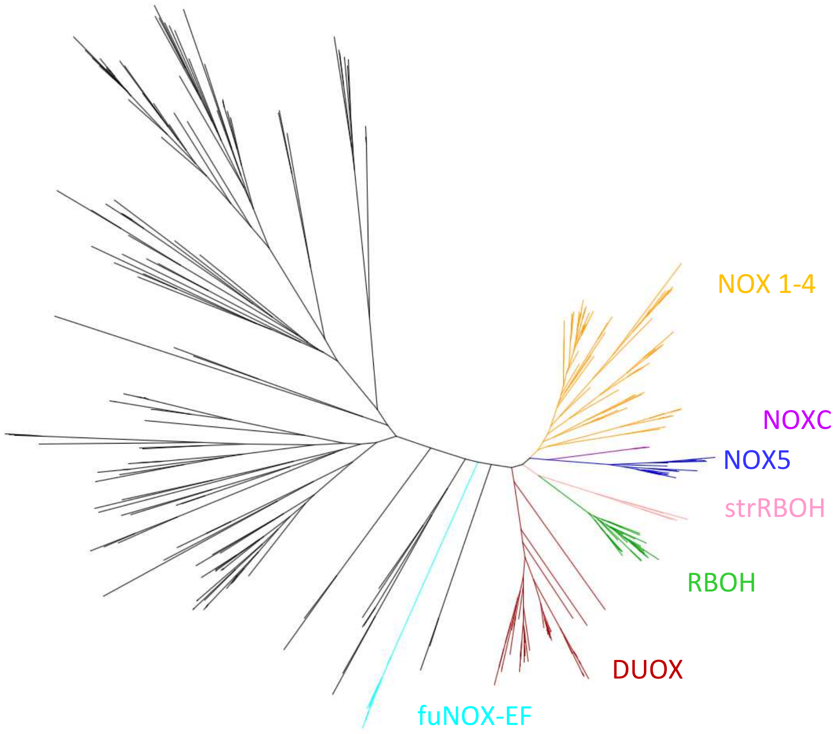

E

ML phylogeny of the FRD superfamily: 308 genes, data model: 215 amino acids, PhyML, WAG+F+gamma(8)+I.

Branch support (aLRT-SH): DUOX: 0.57, NOX5/NOXC: 0.79, fuNOX-ef: 0.48.

Figure S3-4 A-E. ppFRE: A monophyly for the main clade of the ppFRE group is not established; branch support values are indicated. Plant clades (Rhodophyta, Viridiplantae) are colorized green, protist (Euglenozoa, Heterolobosa, Alveolata, stramenopiles) red, fuFRE (Fungi) cyan, Amoeba purple, preFRE golden.

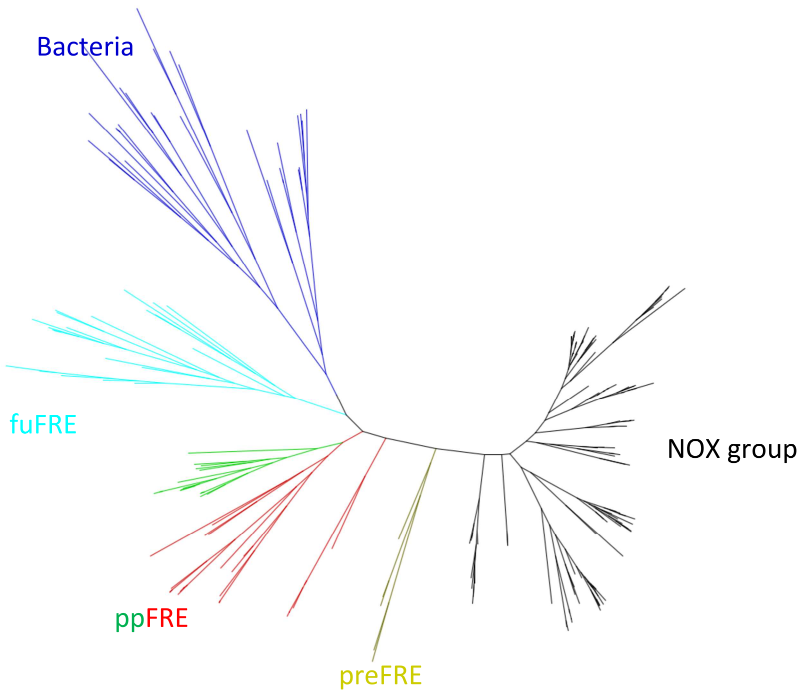

A

BI phylogeny of the FRD superfamily: 198 genes, data model: 171 amino acids,MrBayes, WAG+F+gamma(8)+I, 5m generations.

Branch support, posterior probabilities: fuFRE: 1, ppFRE (main): 0.98; ppFRE (minor): 0.99; preFRE: 1.

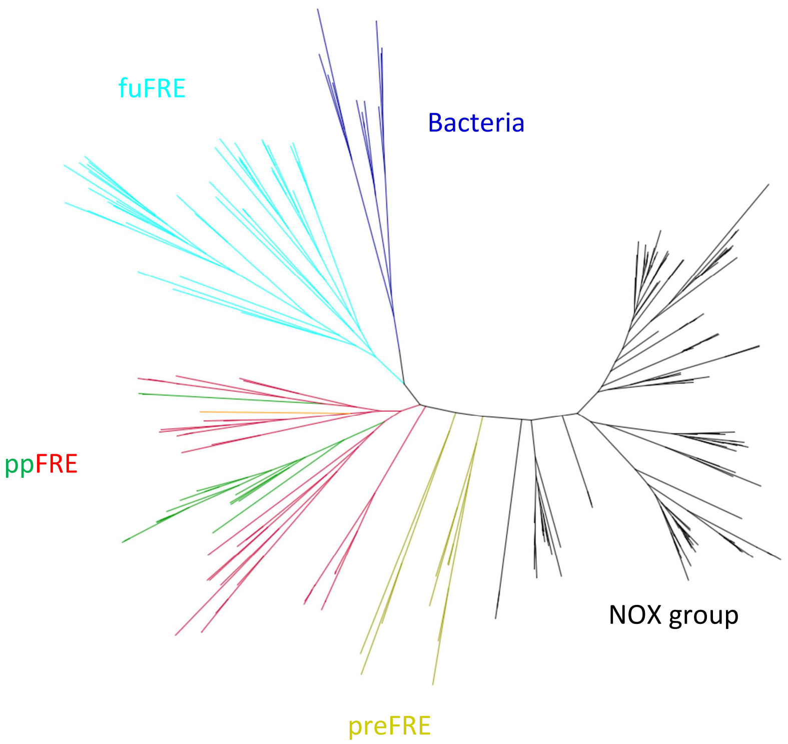

B

ML phylogeny of the FRD superfamily: 240 genes, data model: 156 amino acids, PhyML, WAG+F+gamma(8)+I.

Branch support, SH-aLRT: fuFRE: 0.87, ppFRE (main): 0.87; ppFRE (minor): 0.64; preFRE: 0.87, 0.84.

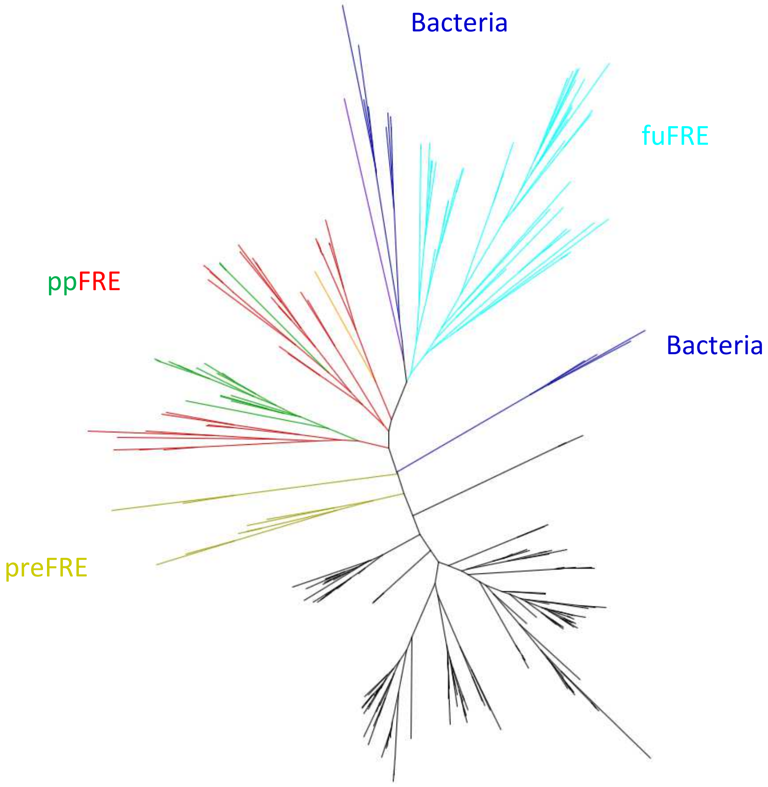

C

ML phylogeny of the FRD superfamily: 240 genes, data model: 156 amino acids, RAxML, WAG+F+gamma(8)+I.

Branch support, bootstrap: very low.

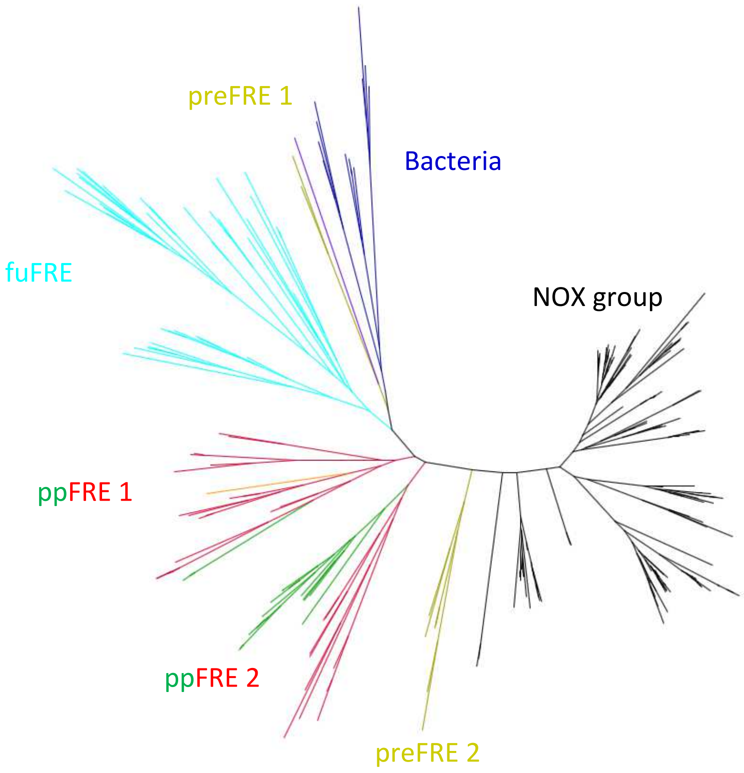

D

ML phylogeny of the FRD superfamily: 240 genes, data model: 156 amino acids, PhyML, LG+F+gamma(8)+I.

Branch support, SH-aLRT: fuFRE: o.93, ppFRE 1: 0.72; ppFRE 2: 0.83; preFRE 1:0.27; preFRE 2: 0.96.

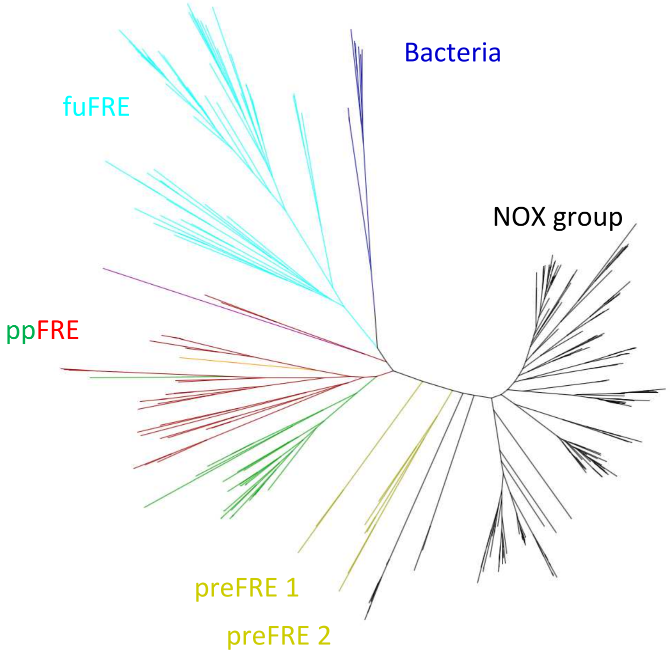

E

ML phylogeny of the FRD superfamily: 308 genes, data model: 215 amino acids, PhyML, WAG+F+gamma(8)+I.

Branch support (aLRT-SH): fuFRE: 0.97, ppFRE (minor): 0.70; ppFRE (main): 0.81; preFRE 1: 0.93; preFRE 2: 0.91.
