# Supplementary material for: Evolution of the Ferric Reductase Domain (FRD) Superfamily: Modularity, Functional Diversification, and Signature Motifs
Source: PLoS One. 2013 Mar 7;8(3):e58126. doi: 10.1371/journal.pone.0058126 (PMC3591440; doi:10.1371/journal.pone.0058126)
Supplement: File S4 — Analysis of bacterial FRD superfamily members. (PDF) [file pone.0058126.s004.pdf]

Figure S4. Analysis of bacterial FRD superfamily members.

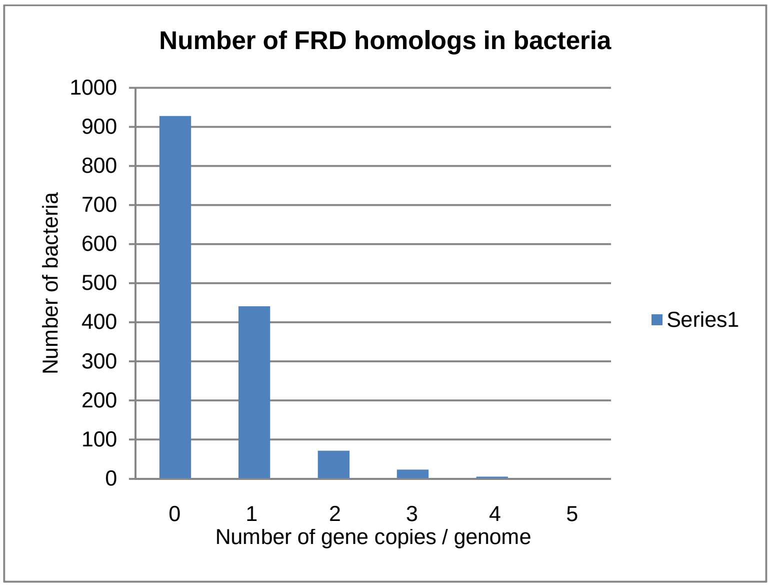

Figure S4-1. Histogram showing the number of FRD superfamily members from 1471 bacteria with completely sequenced genomes. Calculations are based on UniProtKB cross-references to the Pfam domain PF01794.

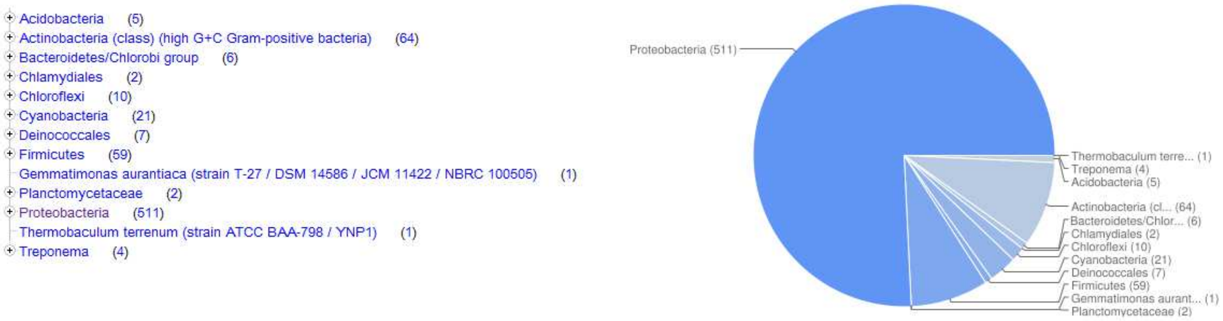

Figure S4-2. Taxonomic distribution of bacteria with completely sequenced proteomes that contain at least one superfamily member (UniProtKB, 29-Feb-2012).

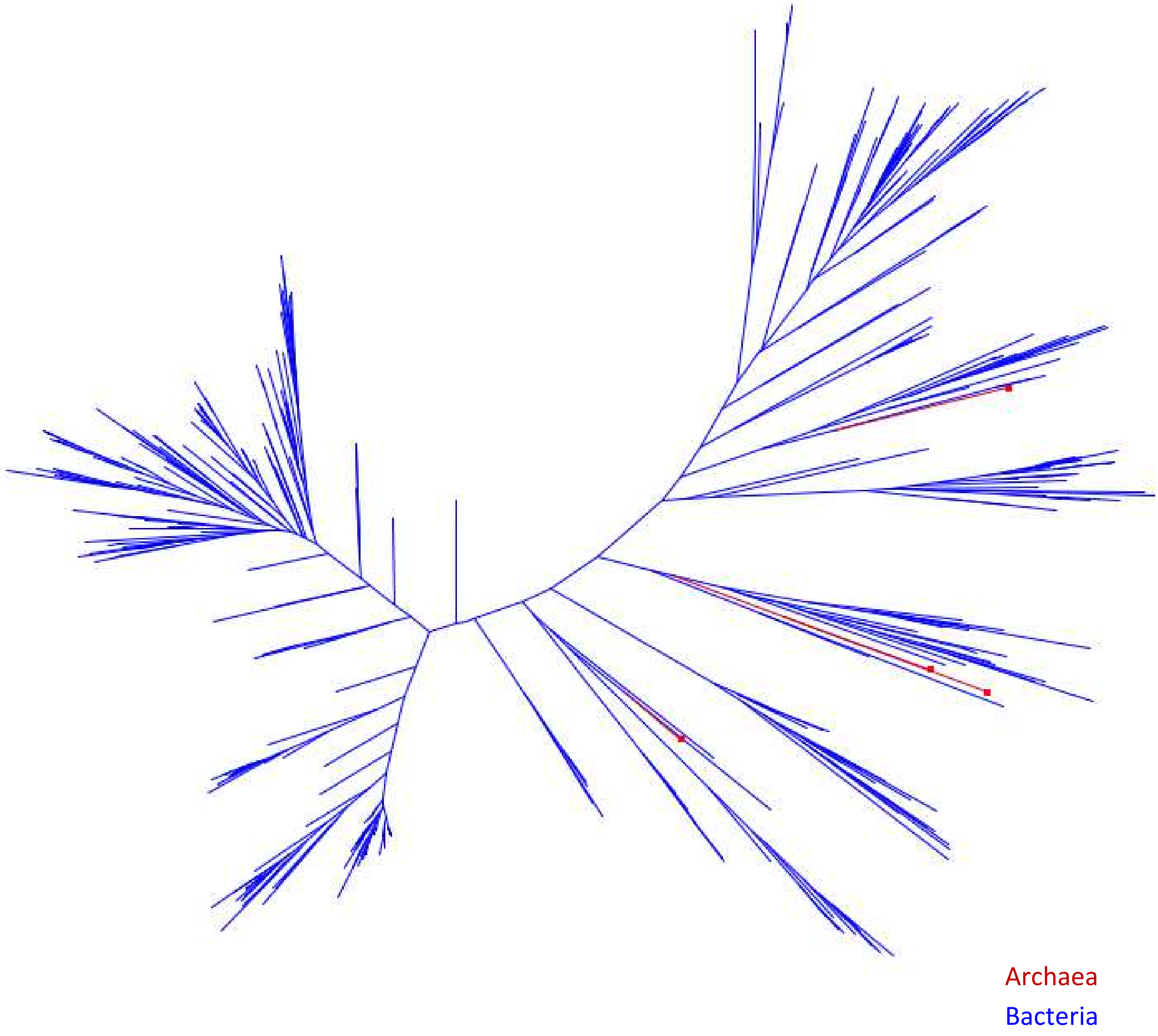

Figure S4-3. ML phylogeny (PhyML, WAG+F+gamma(8)+I) of 565 bacterial FRD superfamily members from completely sequenced genomes and four archaeal homologs. Latter are probably derived by three independent events of horizontal gene transfer.

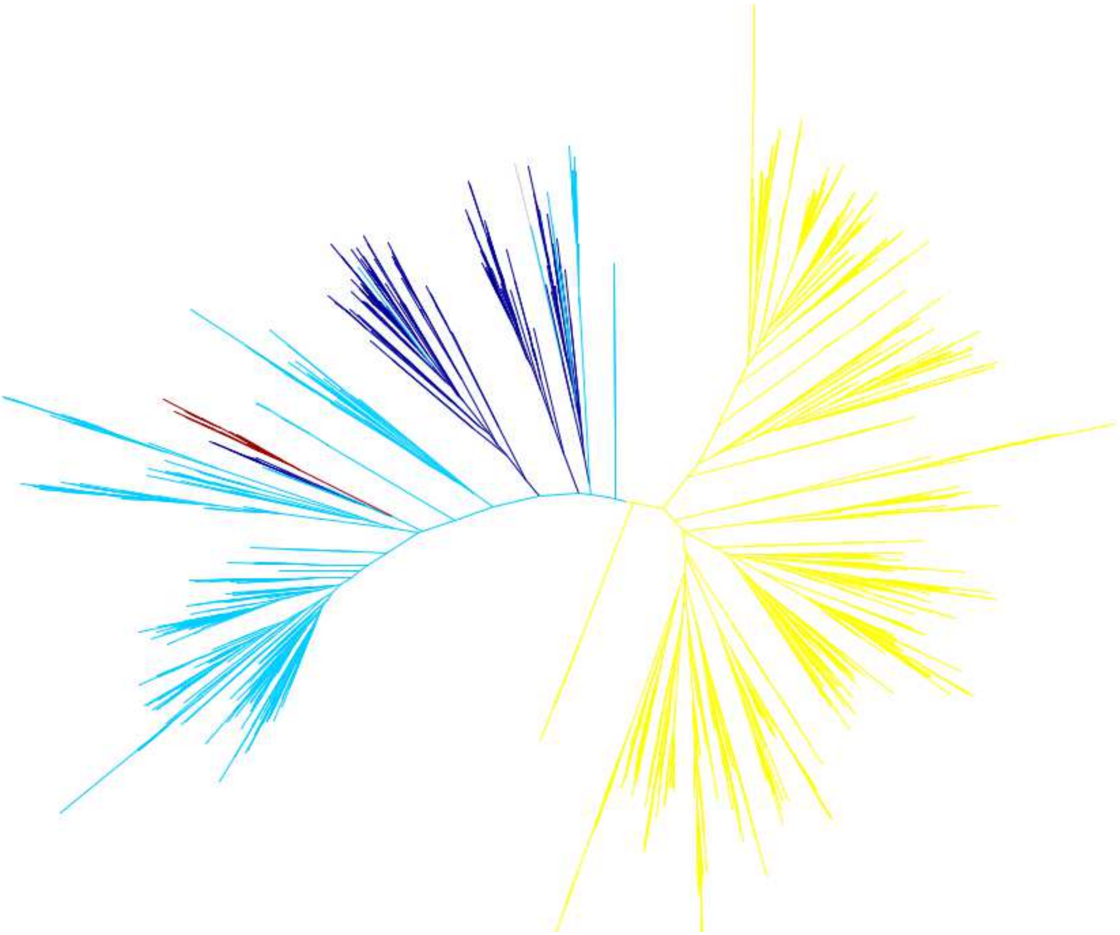

Figure S4-4. ML phylogeny of the superfamily tree including members of the eukaryotic STEAP family (red), bFRD long forms (dark blue), bFRD short forms (light blue), and eFRE and NOX (yellow). The branch support value for the STEAP clade is 0.895.
