# Supplementary material for: Evolution of the Ferric Reductase Domain (FRD) Superfamily: Modularity, Functional Diversification, and Signature Motifs
Source: PLoS One. 2013 Mar 7;8(3):e58126. doi: 10.1371/journal.pone.0058126 (PMC3591440; doi:10.1371/journal.pone.0058126)
Supplement: File S5 — Evolution of the FRD superfamily from the view of its core domains. (PDF) [file pone.0058126.s005.pdf]

File S5. Evolution of the FRD superfamily from the view of its core domains

In order to gather evidence for the origin of the FRD superfamily, we assembled information on dehydrogenase domain-containing proteins that consist of domains predicted by Pfam for FRD superfamily members (Table S5-1). The domain composition was then mapped to the individual phylogenetic domain trees of Pfam 26 (Figures S5, 1-5.).

Domain composition	Hits	FRD (PF01794)	FAD-binding (PF00970)	FAD-binding (PF08022)	NADPH-binding (PF00175)	NADPH-binding (PF08030)
**Bacteria**		**2,531**	**16,299**	**534**	**20,320**	**7**
1	403	+	-	+	+	-
2	3*	+	-	+	-	+
3	73	-	-	+	+	-
4	0	-	-	+	-	+
5	130	+	+	-	+	-
6	0	+	+	-	-	+
7	15,550	-	+	-	+	-
8	2*	-	+	-	-	+
**Eukaryota**		**2,708**	**2,082**	**2,405**	**3,979**	**2,448**
1	5*	+	-	+	+	-
2	1892	+	-	+	-	+
3	0	-	-	+	+	-
4	120	-	-	+	-	+
5	0	+	+	-	+	-
6	0	+	+	-	-	+
7	1615	-	+	-	+	-
8	3*	-	+	-	-	+
**Archaea**		**3**	**197**	**0**	**202**	**0**
1	0	+	-	+	+	-
2	0	+	-	+	-	+
3	0	-	-	+	+	-
4	0	-	-	+	-	+
5	0	+	+	-	+	-
6	0	+	+	-	-	+
7	162	-	+	-	+	-
8	0	-	+	-	-	+

Table S5-1. Domain composition of proteins that possess an FAD-binding and an NADPH-binding domain of the types found in FRD superfamily members. (UniProtKB, Dec-2012). Results that are represented by only a few members (*) might be due to false positives or HGT; they are not considered in the discussion. The search for proteins with specific domain compositions was performed excluding annotated fragments. In contrast, the number of predicted domains for each super-kingdom was determined by including annotated fragments.

The dehydrogenase module is found in multiple protein families of bacteria, eukaryotes and archaea. Both, the FAD-binding domains and the NADPH-binding domains are captured by predictors that belong to the same Pfam clans (CL0076 and CL0091). The most prominent dehydrogenase domain in bacteria is composed of the FAD-binding domain predicted by ‘PF00970’ and the NADPH-binding domain predicted by ‘PF00175’ (Table S5-1). In the bacterial clade of the FRD superfamily tree, the

FAD-binding domain of bFRE long forms is predicted likewise by ‘PF00970’ and ‘PF008022’ (Figure S5-1). In eukaryotes, the dehydrogenase module of FRD superfamily members is predicted exclusively by PF08022 (FAD-binding domain) and PF08030 (NADPH-binding domain). The former seems to be specific for the FAD-binding domain of FRD superfamily members (Figure S5-3), while the latter specifically captures eukaryotic members (Figure S5-5).

Figure S5-2 (FAD-binding domain PF00970 tree) illustrates the likely early emergence of the FRD superfamily in bacteria following the fusion of a ferric reductase domain to the bacterial dehydrogenase module. From the view of the NADPH-binding domain type ‘PF00175’ (Figure S5-4), the bacterial FRD-family members do not form a single clade but seem to emerge from distinct branches. As three independent fusion events are unlikely, alternative explanations would involve the emergence of new families from within the bacterial FRD clade and an erroneous tree topology. A more detailed analysis of phylogenetic trees constructed from all related domains of each clan will help to figure out the most likely evolutionary scenario.

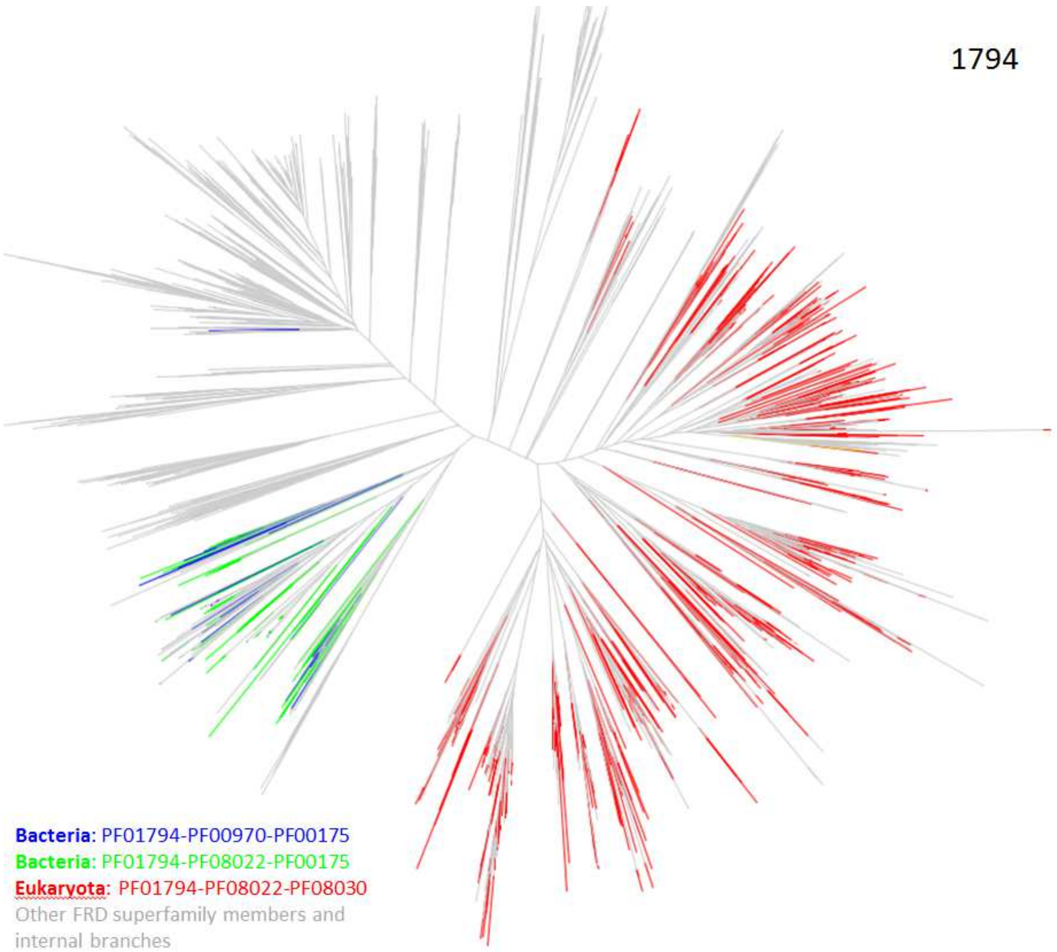

Figure S5-1. Phylogram of the ferric reductase domain (PF01794, Pfam 26.0). Terminal branches are colored according to the proteins' predicted domain composition. The long form of FRD superfamily members are predominantly found in eukaryotes and are less prominent in bacteria. The figure illustrates that the FAD-binding domain of bacterial ferric reductases are predicted by PF00970 (blue) and PF08022 (green). The predominant gray clade includes the bacterial short forms and the STEAP family. Non-colorized terminal branches (gray) within colorized clades can be caused by false negative predictions, incorrect gene models and changes in primary sequence identifiers.

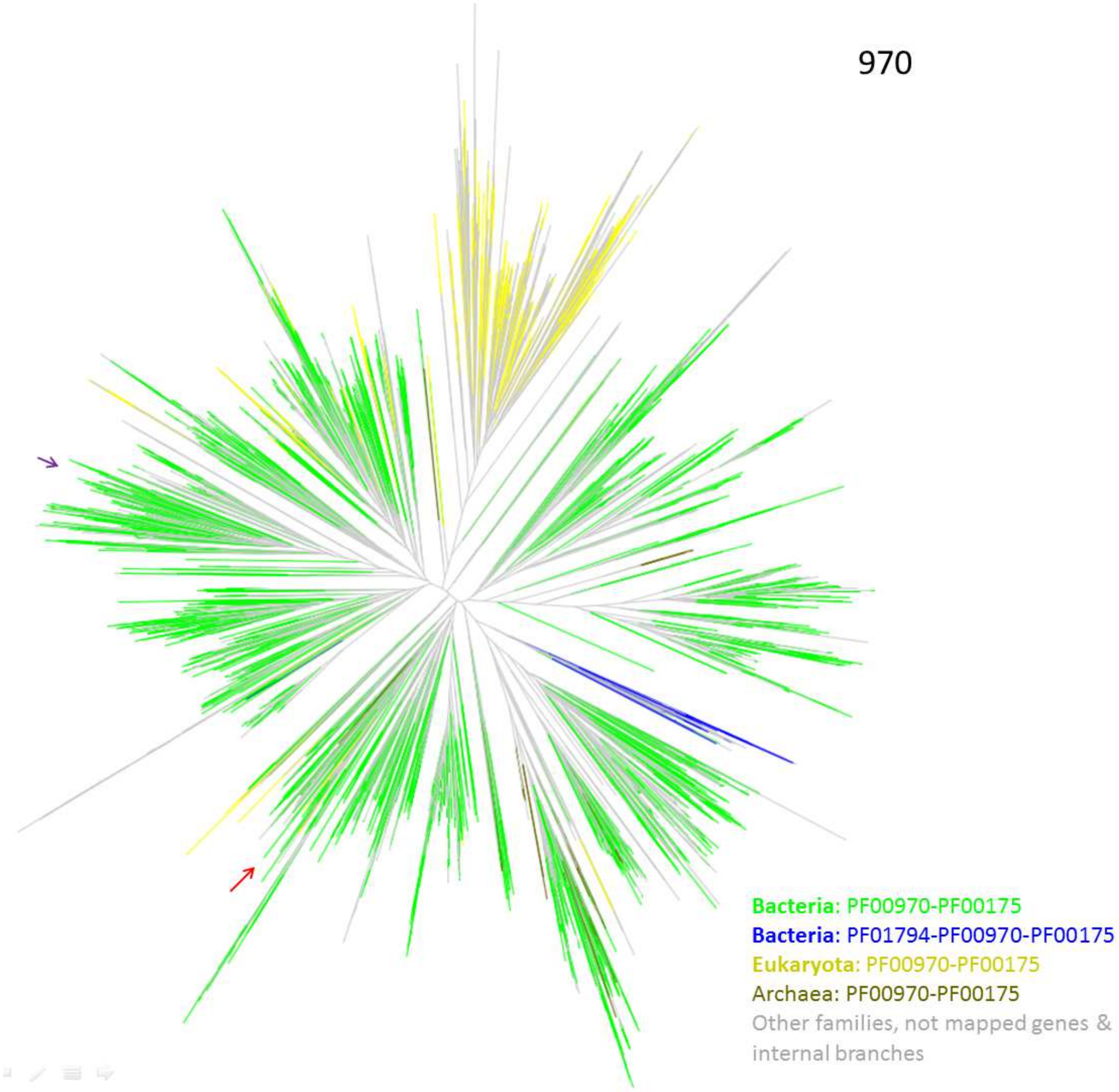

Figure S5-2. Phylogram of the FAD-binding domain type 'PF00970' (Pfam 26.0). This FAD-binding domain generally forms a dehydrogenase module with the NADPH-binding domain of the type 'PF00175'. According to the phylogenetic tree, the bacterial long form of the FRD superfamily (blue) seems to emerge as a monophyletic group early on in the evolution of this protein family via the fusion of a ferric reductase domain. The major eukaryotic clade (yellow) does not include any FRD superfamily members. Of note are other minor eukaryotic clades which are possibly derived via other gene transfer events from bacteria. Examples are the plant ferredoxin--NADP reductases that may have originated from chloroplasts or cyanobacteria (red arrow), and the recent likely gene transfer from Ralstonia metallidurans (Q1LBV0) to Populus trichocarpa (B9PBD6) (purple arrow). Archaeal proteins that possess a dehydrogenase domain (dark gold) do not form a single clade, but rather emerge from multiple branches. For an explanation, see Figure legend S5-1.

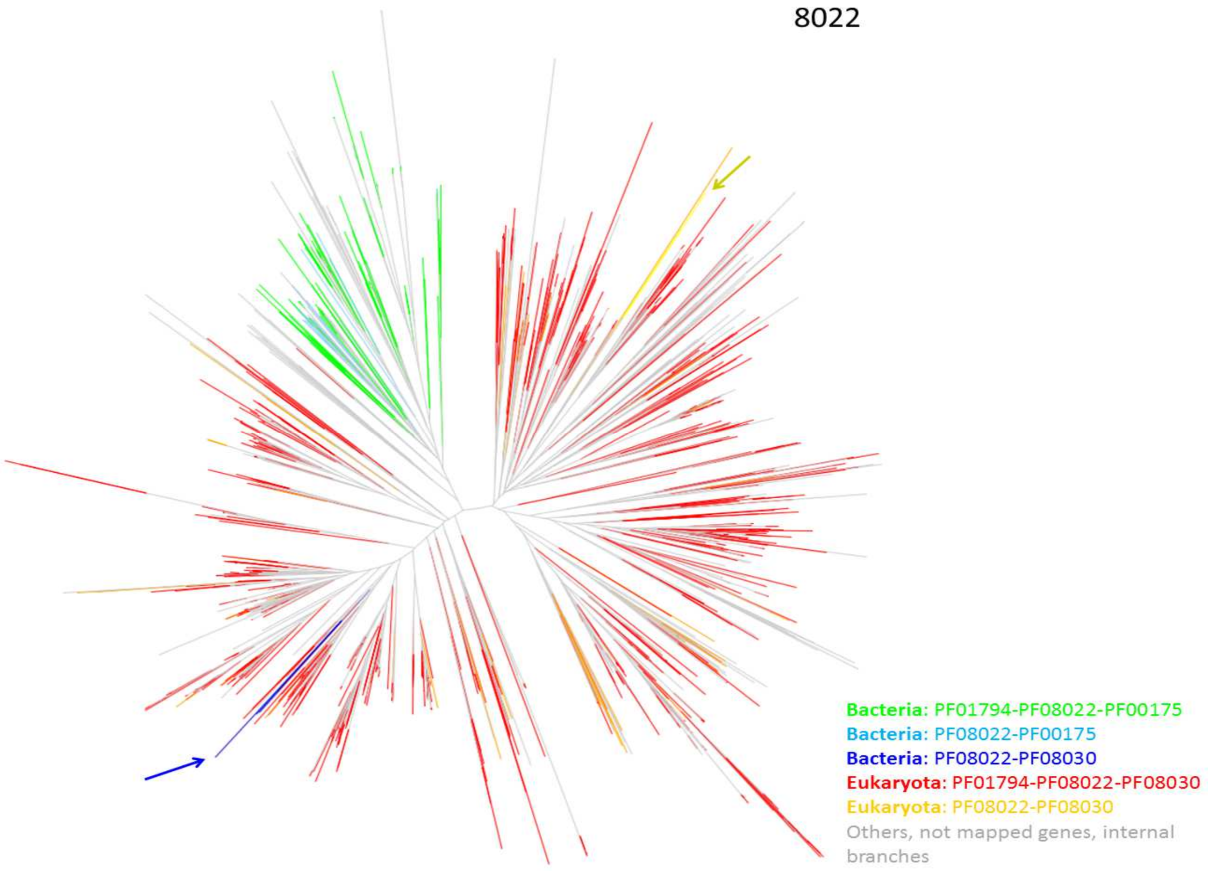

Figure S5-3. Phylogram of the FAD-binding domain type 'PF08022' (Pfam 26.0). This FAD-binding domain seems to be specific to the FRD superfamily dehydrogenase module, as most members possess a predicted ferric reductase domain (red, green). Note that the NADPH-binding domain of the dehydrogenase module is generally of the 'PF00175' type in bacteria (green, blue) and of the 'PF08030' type in eukaryotes (red, orange). An exception in bacteria (blue arrow) probably results from HGT. In contrast to this, the eukaryotic exceptions are probably prediction artifacts (yellow arrow). For an explanation, see Figure legend S5-1.

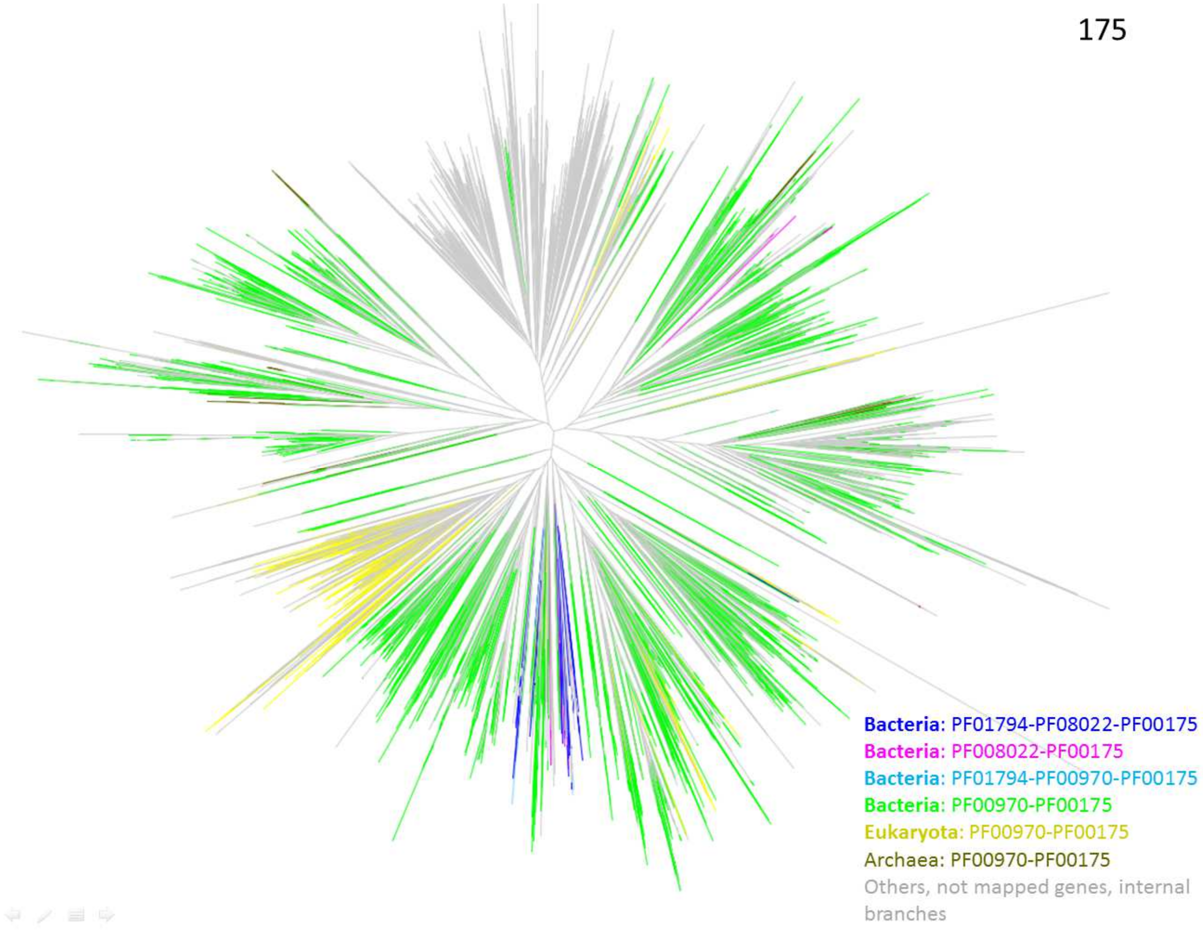

Figure S5-4. Phylogram of the NADPH-binding domain type 'PF00175' (Pfam 26.0). This NADPH-binding domain is found in many bacterial protein families, often as part of a dehydrogenase module together with an FAD-binding domain (green, blue, magenta), mostly of the PF00970 type (green, blue). As shown in Figure S5-1, the FAD-binding domains of bacterial FRD superfamily members (dark blue, blue) are predominantly predicted with PF08022, and relevant clades thus appear in dark blue. In particular, the predicted bacterial ferric reductases form no monophyletic group in this tree. Note that this tree cannot include the typical eukaryotic ferric reductases since their NADPH-binding domains are captured by PF08030. As shown in Figure S5-3, the eukaryotic exceptions (PF01794-PF08022-PF00175) are probably prediction artifacts (red arrow). The largest eukaryotic clade (yellow) includes families such as the nitric oxide synthase (NOS) family and the flavoprotein pyridine nucleotide cytochrome reductase family. Similarly to Figure S5-2, there are several minor eukaryotic clades that may emerge from independent gene transfer events. Archaeal proteins which have a dehydrogenase domain (dark gold) do not form a single clade, but rather emerge from multiple branches. For an explanation, see Figure legend S5-1.

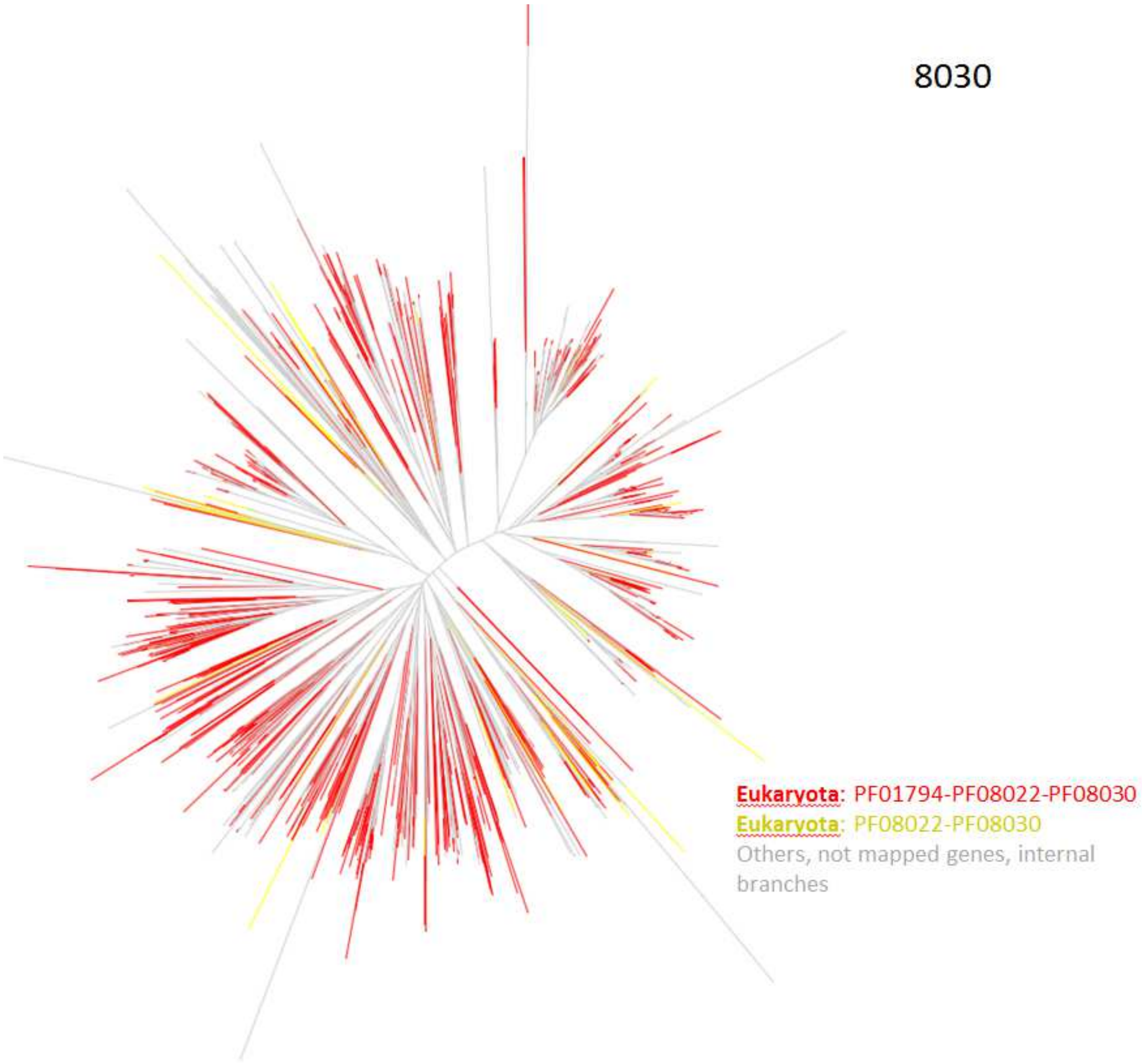

Figure S5-5. Phylogram of the NADPH-binding domain type 'PF08030' (Pfam 26.0). This tree is almost exclusively made up of eukaryotic proteins. According to the predicted domains and the phylogenetic tree, there is no other common and large protein family that includes or consists of a dehydrogenase module of the PF08022-PF08030 type. For an explanation, see Figure legend S5-1.
